# Endophytes in Lignin Valorization: A Novel Approach

**DOI:** 10.3389/fbioe.2022.895414

**Published:** 2022-07-19

**Authors:** Aroosa Jan Mattoo, Skarma Nonzom

**Affiliations:** Department of Botany, University of Jammu, Jammu, India

**Keywords:** endophytic delignification, lignin valorization, recalcitrance, global economy, sustainable development

## Abstract

Lignin, one of the essential components of lignocellulosic biomass, comprises an abundant renewable aromatic resource on the planet earth. Although 15%––40% of lignocellulose pertains to lignin, its annual valorization rate is less than 2% which raises the concern to harness and/or develop effective technologies for its valorization. The basic hindrance lies in the structural heterogeneity, complexity, and stability of lignin that collectively makes it difficult to depolymerize and yield common products. Recently, microbial delignification, an eco-friendly and cheaper technique, has attracted the attention due to the diverse metabolisms of microbes that can channelize multiple lignin-based products into specific target compounds. Also, endophytes, a fascinating group of microbes residing asymptomatically within the plant tissues, exhibit marvellous lignin deconstruction potential. Apart from novel sources for potent and stable ligninases, endophytes share immense ability of depolymerizing lignin into desired valuable products. Despite their efficacy, ligninolytic studies on endophytes are meagre with incomplete understanding of the pathways involved at the molecular level. In the recent years, improvement of thermochemical methods has received much attention, however, we lagged in exploring the novel microbial groups for their delignification efficiency and optimization of this ability. This review summarizes the currently available knowledge about endophytic delignification potential with special emphasis on underlying mechanism of biological funnelling for the production of valuable products. It also highlights the recent advancements in developing the most intriguing methods to depolymerize lignin. Comparative account of thermochemical and biological techniques is accentuated with special emphasis on biological/microbial degradation. Exploring potent biological agents for delignification and focussing on the basic challenges in enhancing lignin valorization and overcoming them could make this renewable resource a promising tool to accomplish Sustainable Development Goals (SDG’s) which are supposed to be achieved by 2030.

## Introduction

Energy demands of exponentially-growing global population are mandatory to be met by available arithmetically-growing resources. Heavy consumption of limited fossil fuels, besides leading to inflation and scarcity of this non-renewable resource, is compromising with the quality and sustainability of the environment (IEA, 2006; Kazi et al., 2010). “To achieve a better and more sustainable future for all”, Sustainable Development Goals (SDG’s) were set by United Nations General Assembly in 2015 with the introduction of 17 inter-connected goals to be achieved by 2030. Preferably, to accomplish SDG 7 “Affordable and clean energy” and SDG 13 “Climate action”, renewable, eco-friendly, and sustainable alternatives, such as, lignocellulose capable of conversion into biofuels, are imperative for satisfying global demands (Brodin et al., 2017; Wang et al., 2020).

Lignocellulose is the major renewable biomass resource on the earth consisting of an average 40%–50% cellulose, 25%–30% hemicellulose, and 15%–20% lignin (Alvira et al., 2011; Chaurasia, 2019). Cellulose pertains to the chief polysaccharide of the lignocellulose in which glucose monomers are assembled via β-1, 4 ether linkages giving rise to linear unbranched chains. Hemicellulose, on the other hand is a heteropolymer consisting mainly of xylans and mannans (Mahmood et al., 2016). Its branched heterogeneous polysaccharides consists of pentoses, like, xylose and arabinose; hexoses, like, glucose, galactose, and mannose; and sugar acids, like, galacturonic, glucuronic, and acetic acids (Dimarogona et al., 2012). Hemicellulose intertwins with both cellulose and lignin to form a network in plant cell wall.

Lignin is regarded as the most abundant aromatic biopolymer present in the vascular tissues (xylem tracheids, vessels) and sclereids of most of the plants to provide the mechanical support (Glasser, 2019). Being hydrophobic in nature, it resists the water absorption through plant cell wall. Lignin has an immense potential to be used in the production of biofuel and numerous valuable chemicals (Vavilala et al., 2019). Every year, millions of tonnes of lignin produced in paper and pulp industry is considered as a low-value byproduct thereby utilized in heat generation (Demuner et al., 2019). It means besides downgrading the importance of an abundant and valuable resource, ecologically unhealthy practice is being employed. Therefore, it is imperative to valorize this resource as per its potential using eco-friendly technologies.

Although lignin can efficiently be used as an alternative energy resource to fossil fuels and diverse valuable chemicals (Vavilala et al., 2019), its predominant linkage with the hemicellulose, structural complexity, heterogeneity, insolubility, and stability poses difficulty in its application (Lu et al., 2017). Valorization of lignin requires its separation from the lignocellulose followed by depolymerization. Diverse technologies are being developed at various scales to depolymerize this complex biopolymer. Much attention has been given to the thermochemical techniques owing to their long history of application in lignin deconstruction (Pandey and Kim, 2011). However, these methods are costly, require high energy, yield undesirable and variable products, and are not eco-friendly (Iram et al., 2021). In the recent years, biological methods have gained much attention of the researchers owing to their environment friendly and cost-effective approach, along with their exclusive ability of funnelling heterogeneous intermediates into specific desirable products (Li and Zheng, 2020).

In nature, innumerable microorganisms from diverse sources possess inherent capability to degrade lignocellulose. This ability is attributed to the potent lignin modifying enzymes (LME’s) produced by these microbes (Weng et al., 2021). Out of these microorganisms, endophytic microbes are being projected as one of the potent delignifiers (Fillat et al., 2017). Endophytes are the ubiquitous microbes, such as, bacteria, fungi, actinomycetes that reside asymptomatically within the plant tissues, atleast for a fraction of their lifecycle (Bacon and White, 2000). Mostly mutualistic in nature, these microbes confer a plethora of advantages to the host plants, acquiring food and shelter in return (Mattoo and Nonzom, 2021). However, they are highly dynamic and can switch their lifestyle to saprophytism or pathogenism, either in specific stressful conditions or after the death of their hosts (Schulz and Boyle, 2005). After host senescence, endophytes are the pioneers to breakdown its (hosts) complex polymers into monomeric forms (Promputtha et al., 2007; Parfitt et al., 2010; Szink et al., 2016). Therefore, this microbial group can furnish an exciting and fruitful frontier to be explored that may broaden our vision towards lignin depolymerization, unveil novel enzymes, novel pathways, thereby helping in the construction of efficient biorefinaries. The contemporary review desires to dispense insights into the challenges in lignin depolymerization with the brief comparative account of thermochemical and biological methods followed by emphasis on the capabilities of endophytes in lignin deconstruction and valorization into microbial lipids, vanillin, polyhydroxyalakanoates, and *cis-cis* muconic acid. Furthermore, few optimization techniques recently employed to enhance the lignin degrading potential of endophytes are also being highlighted.

### Complexity and Heterogeneity of Lignin: A Challenge for Depolymerization

**FIGURE 1:**
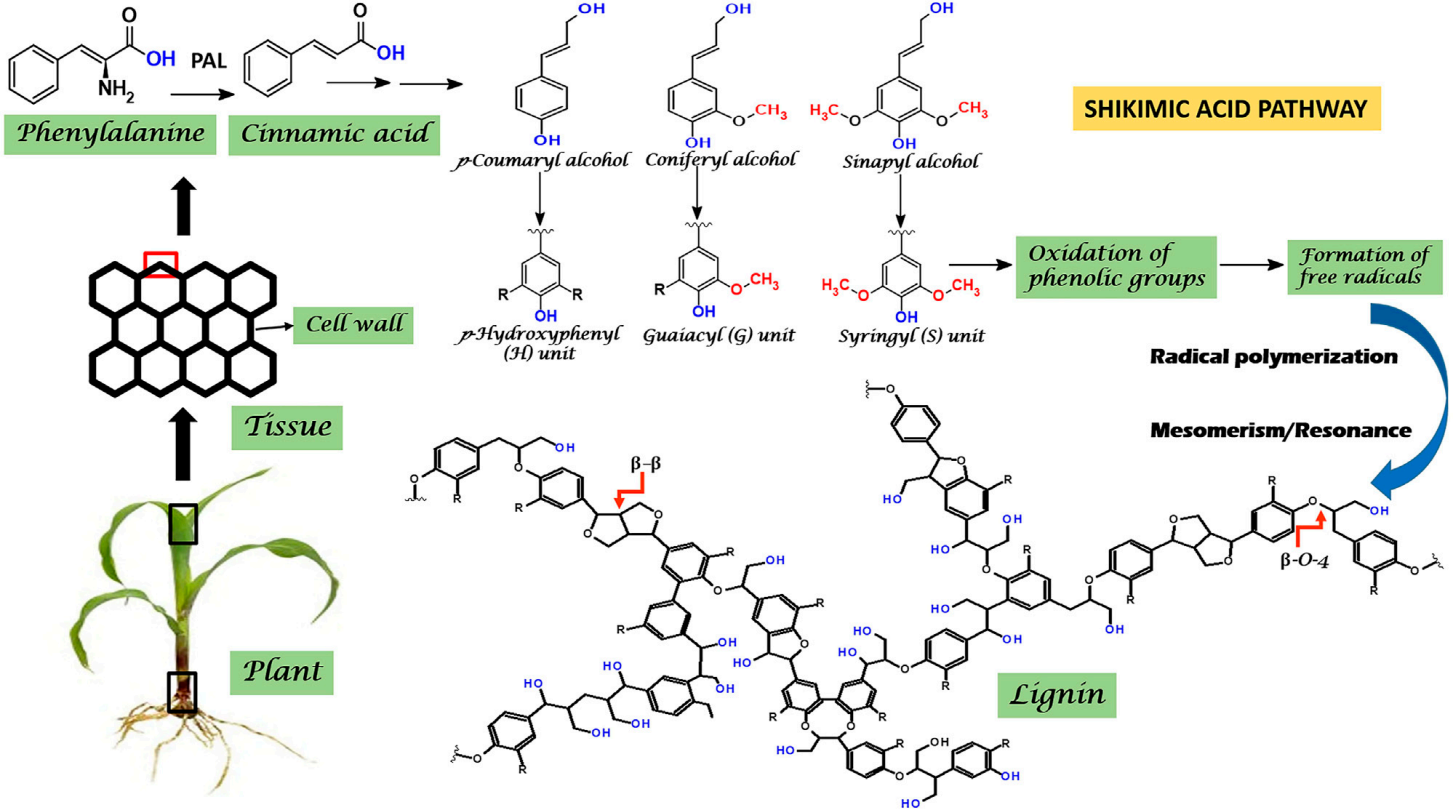
Schematic representation of Shikimic acid pathway involved in lignin biosynthesis. Where PAL, Phenylalanine lyase.

Lignin is an amorphous, highly branched, three-dimensional, complex heterogenous polymer with both phenolic and non-phenolic components (Lu et al., 2017). Chemically, it is formed by the radical polymerization of three phenylpropanoid monomers, namely, guaiacyl units (G), syringyl units (S) and p-hydroxyphenyl units (H), the derivatives of precursor hydroxycinnamyl alcohols (monolignols) viz., coniferyl, sinapyl, and *p*-coumaryl alcohols, respectively (Ralph et al., 2004) (Figure 1). Lignin monomers could be interconnected through carbon-carbon bonds (β-1, β-β, β-5, 5-5) and ether bonds (β-O-4, 4-O-5), however, β-O-4 comprises the most predominant linkage in lignin biopolymer (Boerjan et al., 2003; Alcalde, 2015).

Biosynthesis of lignin monomers occurs through the Shikimic acid pathway (Higuchi, 1990). Initially, the enzyme phenylalanine lyase (PAL) catalyzes the conversion of phenylalanine into cinnamic acid. A multistep process thereby led to the formation of three basic monolignols from cinnamic acid. The phenolic groups of the monolignols undergo an enzymatically catalyzed oxidation reaction (dehydrogenation) giving rise to the free radicals (Ralph et al., 2004). Simultaneously, the radical coupling reaction takes place on account of the unstable nature of free radicals to form lignin. Interestingly, monolignols being aromatic alcohols contains the conjugated single-double bonds in the benzene ring which remains in a state of mesomerism/resonance. This property couples the monolignols in multiple ways, changing the number and nature of bonds in the final structure of lignin polymer, hence a prominent cause for lignin heterogeneity (Figure 1). Depending on the type of lignocellulosic biomass, the amount of monolignols vary (Liu et al., 2018). Lignin mainly comprises of coniferyl (G) units in softwood (90%–95%), and sinapyl (S) units in hardwood (45%–75%), while significant amount of p-coumaryl (H) derived units are found in grasses (5%–35%) which has lesser quantity in both hardwoods and softwoods (Gellerstedt and Henriksson, 2008; Lourenco and Pereira, 2018).

On account of the complex chemical bonding between its monomers, lignin shows low biodegradability which has become a great challenge for its valorization. The main issue lies in its natural heterogeneity that hinders the formation of common valuable target products (Cao et al., 2018). To convert this abundant and renewable resource into valuable outputs, cooking (removal of lignin from lignocellulosic biomass) followed by depolymerisation (conversion of lignin polymer into oligomers and monomers) is the basic requirement. As already discussed, most of the lignin is produced as a by-product in pre-treatment of lignocellulose in the pulp and paper industry. It is called technical lignin on account of the differences in its structure and composition from the natural lignin (Achyuthan et al., 2010; Kazzaz and Fatehi, 2020). This difference lies in the pre-treatment methods applied for the separation of lignin from cellulose and hemicellulose, the process called cooking. The latter is required to solubilize the otherwise insoluble native lignin, thereby facilitating its separation. Traditionally, two methods are known for cooking—Kraft cooking method and Sulfite cooking method, former being the primary pulping process. In Kraft process, substrate is treated with the aqueous solution of sodium hydroxide (NaOH) and sodium sulphide/white liquor (Na2S) at high temperature (150°C–180°C). In Sulfite process, the reaction can take place in acidic, neutral or alkaline solution. Here, hydrolytic cleavage of ether bonds is followed by sulfonations via sulfite ions (Lu et al., 2017). The lignosulfonates so formed are water soluble. Recently, Alcell and Organocell methods are being introduced (Abdelaziz et al., 2016). Although the formation of Kraft and Sulfite lignin is economical, the unwanted sulphur and hemicellulose content alongside the target products with their less solubility in organic solvents constitutes the main disadvantages (Abdelaziz et al., 2016). On the other hand, Organosolv lignin is devoid of these limitations but the cost is high (Abdelaziz et al., 2016).

## Methods of Lignin Depolymerization

**FIGURE 2:**
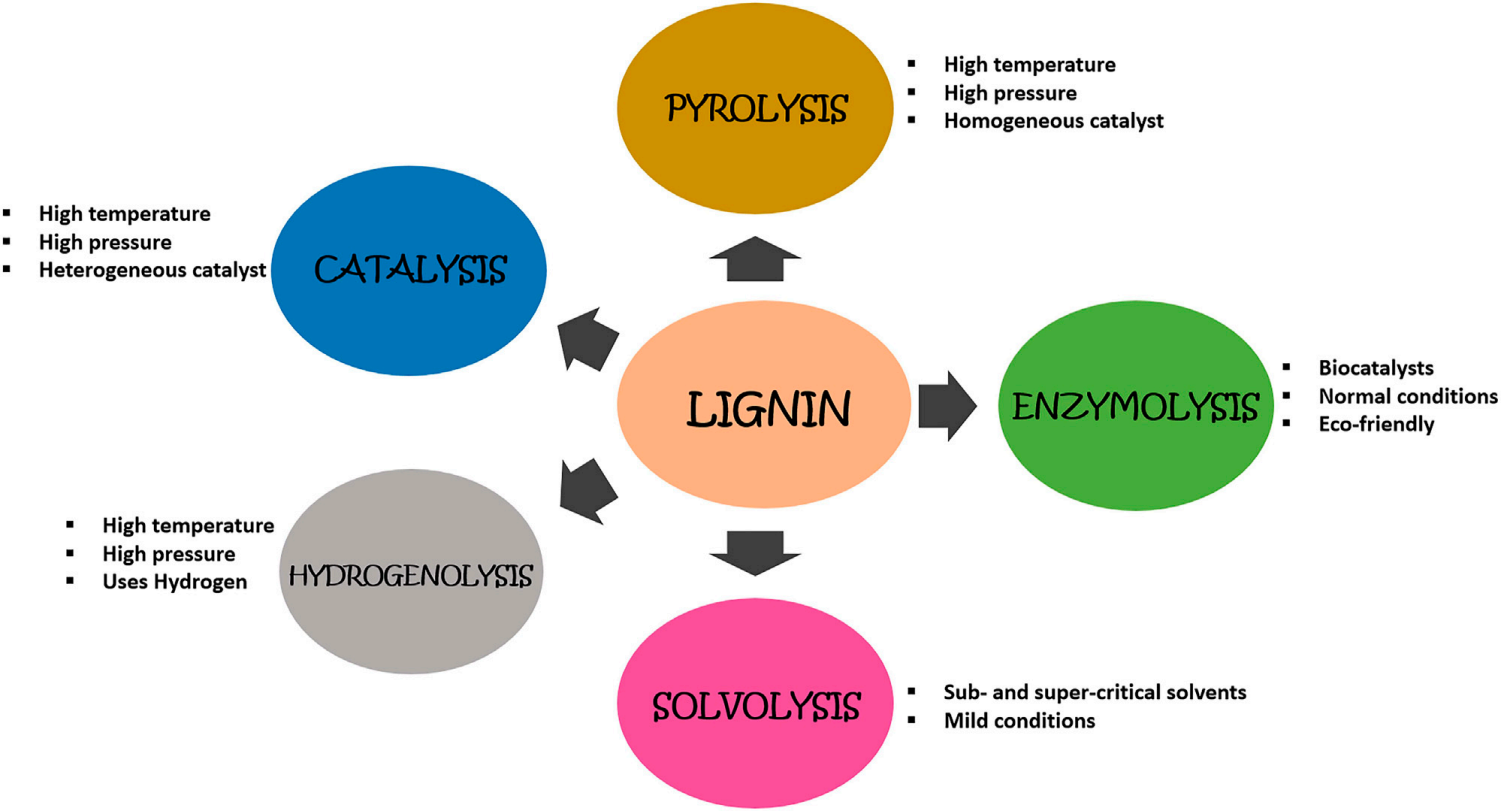
Diagrammatic representation of different methods employed to degrade lignin biopolymer.

Depolymerization of lignin pertains to its degradation into oligomers and monomers which could subsequently be utilized to generate valuable products. Industrially, this is the principal step applying various physical, chemical, and biological methods to depolymerize this recalcitrant biopolymer (Figure 2). Characteristic features of various delignification techniques are also mentioned in Table 1. This process requires the breakage of bonds linking different components of lignin, mainly β-O-4 linkages. Different methods which are employed to depolymerize lignin are as follows:

**TABLE 1:** Comparative analysis of diverse methods used in lignin deconstruction.

S. No.	Parameter	Pyrolysis	Catalysis	Hydrogenolysis	Solvolysis	Enzymolysis
1	Temperature requirement	High	High	High	Relatively less	Less
2	Pressure requirement	High	High	High	Relatively less	Less
3	Catalyst type	Homogeneous	Heterogeneous	Heterogeneous	Heterogeneous	Enzymatic
4	Prediction of outcome	Least	Least	Less	Less	More
5	Repolymerization	More	More	Comparatively less as hydrogen removes radicals formed during the reaction	Happens, high temperature required to lessen repolymerization	None or meagre
6	Desirability/selectivity of product	Less	Less	Less, requires different conditions with higher energy	Comparatively more	High
7	Variability of outcome	High	High	High	High	Less, involves biological funnelling
8	Cost	Costly	Costly	Costly	Costly	Economical
9	Influence of lignin source	High	High	High	Comparatively less	Less
10	Environment friendly and clean process	No	No	No	No	Yes

### Thermochemical Delignification

#### Pyrolysis and Co-Pyrolysis

It is an endothermic reaction which requires high temperature and inert environment to degrade an organic compound (He et al., 2006). Delignification via pyrolysis involves the degradation of lignocellulosic biomass/lignin at the temperature of 300°C–600°C, with/without a catalyst in the absence of oxygen (Huber et al., 2006). Essentially, high temperature cleaves hydroxyl (OH) functional group linked to aliphatic side chain and mainly led to the formation of alkanes, alkenes (mostly with C2 and C3), phenolic compounds, toluene, *p*-Xylene, etc. At times, the linkage between aromatic rings formed after cleavage forms the mixture of catechols, guaiacol, syringol, and phenols (Ghysels et al., 2020). The yields of desirable and undesirable products are affected by the methods employed. For instance, different types of lignin upon treatment with Py-GC/MS and TGA/FTIR techniques in the catalytic and non-catalytic pyrolysis detected >50% volatile compounds (Zhang et al., 2012; Zhang et al., 2014). However, the least desirable products were obtained from the Kraft lignin, the most common cooking method in the paper industry. It indicates that cooking process significantly influences the outcome of pyrolytic lignin degradation. Moreover, this method yields variable compounds and the nature of compounds vary not only with respect to cooking methods but also with the source of lignin and the catalysts utilized. It has been observed that catalysts have the ability to modify the outcome of the pyrolytic reaction. For example, introduction of zeolite catalysts enhanced the yield of toluene and *p*-Xylene (Yu et al., 2012). Also, apart from more energy requirement, many reactions exhibit reversibility. For example, Bai et al. (2014) detected the significant fraction of phenolic oligomers in bio-oil during the pyrolytic delignification, which basically were the outcome of re-polymerization of phenolic monomers. In this way, one cannot predict the exact composition of target products which is a major problem to achieve a particular target. High temperature and more energy requirement, melting issues, and re-polymerization of monomers along with the lesser prediction of outcome are the main difficulties associated while performing pyrolysis of lignin.

In the recent years, many efforts are being made to overcome these difficulties. To cope up with the melting and re-polymerization, various additives have been tested. For example, addition of the calcium hydroxide, calcium formate, and clay (attapulgite, bentonite and sepiolite) to lignin were found to give promising results (Ghysels et al., 2020). In addition, lignin-attapulgite was also found to be the most promising additive with highest yield of carbon and cresylic acid (phenol, cresols, xylenols). Similarly, lignin-calcium carbonate mixture yielded the highest quantity of monomeric lignin, and prevented their re-polymerization into oligomers (Ghysels et al., 2020). Likewise, synergistic effects of lignin with waste materials especially plastic is being promoted to enhance the quality of certain lignin-derived chemicals. It not only improves the monomeric yield of lignin but also helps in recycling of non-biodegradable wastes. In an attempt, lignin was co-pyrolyzed with polyethylene and polystyrene at a temperature of 500°C. With red clay as catalyst, GC-MS/FID analysis showed the effective depolymerization of lignin into guaiacol, a monomeric derivative of lignin (Patil et al., 2018). Similarly, co-pyrolysis of 1, 4-butanediol with lignin in a microwave reactor enhanced the production of selected monomeric phenols to 3-fold (Tarves et al., 2017). When lignin and cellulose biopolymers were co-pyrolyzed with plastic wastes (polyethylene and polystyrene) with 10 wt% nickel supported on MCM-41 as catalyst, a hydrogen-rich syngas was obtained (Akubo et al., 2020). Comparative analysis of Ni/MCM-41 catalyst with Ni/Al_2_O_3_ and Ni/Y-zeolite-supported catalysts showed that Ni/Al_2_O_3_ catalyst exhibits the higher product yield. Apart from plastics, waste rubber has also been used as a co-polymer during co-pyrolysis using zeolite catalyst of NaY to produce hydrocarbons and phenols (Cui et al., 2020). Product analysis was done employing thermo-gravimetric analyzer (TG) and pyrolysis-gas chromatography/mass spectrometry (Py-GC/MS). Similarly, co-pyrolysis of lignin with sawdust produced biochar, a carbonaceous liquid (Li et al., 2021). Also, a varied aspect of co-pyrolysis has recently came into limelight when an eco-friendly C–Ni/Al_2_O_3_ composite was prepared during the catalytic reforming of volatiles derived from co-pyrolysis of lignin and polyethylene by depositing carbon on a Ni/Al_2_O_3_ catalyst (Zhao et al., 2021). Although co-pyrolysis is considered as a promising technique for converting solid wastes into value-added products with the involvement of catalysts to further enhance the conversion efficiency of feedstock selectivity to target products (Xu et al., 2021; Li et al., 2021), it is costly and leads to the environmental pollution.

#### Cracking

This process involves the breakdown of a material by heating it under pressure in the presence of catalysts. Differing from pyrolysis in the presence of a heterogeneous catalyst, cracking can function with or without hydrogen. Heterogenous catalysts, apart from enhancing the reaction rate, can a bit channelize the reaction towards the common target. For example, application of iron oxide to the delignification reaction selectively yielded phenolic compounds viz., methoxyphenols and catechol (Yoshikawa et al., 2014). Selective conversion of lignin to ethylbenzene was accomplished by the process of thermal cracking using Ni/Silicalite-1 (Ni/S-1) as a heterogenous catalyst (Luo et al., 2020). Here, lignin was hydrodeoxygenated to C_8_ ethylcyclohexane, C_9_–C_17_ cyclic alkanes, and few C_3_–C_7_ alkanes of gasoline range using Ni/Silicalite-1 (Ni/S-1) catalyst at 300°C and 6 MPa H_2_ in the presence of a non-polar solvent. Thermal cracking proceeds through the elimination of C_β_–C_γ_ bonds in the propanyl chain of depolymerized C_9_ radicals, similar to the homolytic cleavage of β-O-4 linkages in lignin quinone methide reaction pathway. Subsequently, Ni NPs facilitate decarboxylation of carbonyl groups resulted from ester and hydroxyl groups of C_9_ lignin units.

Traditional cracking involves the application of harsh conditions (high temperature, high pressure) to break the complex and stable bonds of lignin polymer. However, recently, an ionic liquid system has been devised which involves the use of ethyl ammonium nitrate (EAN) and prolinium tetrachloromanganate (II) [Pro]_2_[MnCl_4_]. The noteworthy aspect of the reaction pertains to the application of low temperature (35°C) and atmospheric pressure as the reaction conditions (Mehta et al., 2021). On account of higher solubility of lignin in ethyl ammonium nitrate, maximum number of H-bonding sites are exposed that lead to cracking of otherwise recalcitrant lignin, followed by oxidative conversion by [Pro]_2_[MnCl_4_] through the bonding between Mn and lignin biopolymer. Confirmation of delignification was done by SEM, FT-IR, PXRD, and GC-MS analyses.

Being a hub of hydroxyl groups, lignin is highly susceptible to photocatalytic cracking. As per an investigation, lignin obtained through two pulping processes—organosolv and Bmim (MeSO_4_) pulping was treated with titanium oxide (TiO_2_) under UV light. GC-MS analysis revealed the identity of main degradation products as syringaldehyde, pyrocatechol, and raspberry ketone after treatment with UV light for 1 h (Prado et al., 2013). The main drawback of the study was the char formation which did not show significant variation with the photochemical treatment. Various insights into the mechanism of photocatalysis are being made using the most dominant linkage *β-*O-4 dimer. However, TiO_2_ technology for the degradation of a *β-*5 model dimer involving photocatalysis recently came into limelight (Murnaghan et al., 2021). Besides, TiO_2_, metal sufide photocatalysts are also gaining essence (Wu et al., 2021).

Recently, special attention was paid to photocatalysis in terms of enhancing the monomeric yield of lignin. For instance, two novel iso-propylamine-based lead chloride perovskite nanomaterials (SK9 and SK10) synthesized by the facile hydrothermal method were investigated for photocatalytic delignification under UV light (Kausar et al., 2020). Further, characterization employing Powder X-Ray Diffraction (PXRD), Scanning Electron Microscopy (SEM), UV-Visible (UV-Vis), Photoluminescence (PL), and Fourier-Transform Infrared (FTIR) and GC-MS revealed the synthesis of 2-methoxy-4-methylphenol (39%), benzene (17%), phenol (10%) and catechol (7%) as the main degradation products. However, the enhanced production of these monomeric units was favoured by high temperature. Although variable products can be formed by degrading lignin under variable conditions, the percentage of selectivity is quite less. In an attempt, almost complete photocatalytic delignification was achieved by adding carbon nanodots decorated TiO_2_ nanohybrid in aqueous conditions under direct sunlight (Sharma et al., 2020). By this method, a different variety of lignin derivatives, such as, m-anisic acid (3-methoxybenzoic acid) and p-hydroxybenzoic acid (PHBA) were formed with a selectivity percentage of 20% and 16.25%, respectively. Therefore, advanced cracking techniques may help degrading the lignin at relatively milder conditions.

#### Hydrogenolysis

Degradation of a material in the presence of hydrogen or reductive degradation of a material is called hydrogenolysis. During this process, hydrogen is added at a high temperature and pressure. As reduction either involves the addition of hydrogen/electrons or the removal of oxygen, reaction involving both the addition of hydrogen and removal of oxygen is termed hydrodeoxygenation. Both the hydrogenolysis and hydrodeoxygenation act as promising methods of delignification, comparatively offering better yields and selectivity of the products (Shu et al., 2020). During this process, the predominant ether bonds can be readily cleaved (Li et al., 2020).

Traditional hydrogenolysis had various issues regarding the high energy requirement, low yield, less prediction towards the synthesis of desirable products and so on. However, attempts are being made to enhance the efficiency of hydrogenolysis through the introduction of diverse catalysts. Catalysts exhibit remarkable impact on the production of selective monomers as the lignin depolymerizaion products. For instance, NiAu catalyst assisted hydrogenolysis of organosolv lignin yielded 14 wt% aromatic (phenolic) compounds at low temperature (Zhang et al., 2014). Intriguingly, in a reductive processing of birch wood, OH-content of the lignin derived phenolic products was drastically enhanced after replacing Ru/C with Pb/C catalyst (Van den Bosch et al., 2015). In another study, continuous hydrogenolyis of acetal-stabilized lignin isolated by pretreatment of birch particles produced 45% monophenolic monomers in a batch reaction with Ni/C at 200°C for 15 h (Lan et al., 2021). With the excess of catalyst, stable depolymerisation was observed in steam for 125 h. Also, using Ru/C catalyst for continuous hydrogenolysis, the monomeric yield reached upto 40% after 20 h which remained stable upto 80 h. The dominant products formed were propylphenols and phenylpropanols with the selectivity of 65% and 33%, respectively (Lan et al., 2021). Similarly, C-lignin, a homo-biopolymer exclusively made up of caffeyl alcohol acts as the most efficient feedstock for the formation of catechol dervatives. It has been observed that hyrogenolysis in the presence of an atomically dispersed Ru catalyst broke the C−O bonds in benzodioxane linkages of C-lignin to form catechols in high yields. Also, unique 77% selectivity was observed in propenylcatechol synthesis (Wang et al., 2021). The metal catalysts accelerate the β-O-4 cleavage and stabilizes the hydrogenation products, thereby accounts for the continuous and selective synthesis of target compounds.

Catalytic hygrogenolysis in the presence of non-aqueous solvents also proved to be effective. Organosolv poplar lignin (OPL) upon hydrogenolysis using an array of nickel-copper catalysts produced valuable monophenols (Cheng et al., 2021). Both the monomeric yield and selectivity was better. Also, to maximize the efficiency of monomeric yield, normal solvents were replaced with super critical solvents. For instance, alkali lignin in super critical ethanol was catalytically added with hydrogen in the presence of copper monometallic catalyst supported on a chromium-based metal organic framework (Tran et al., 2021). The depolymerized products mainly composed of G-type monomers which act as the favourable feedstock for microbial bioconversion. Similarly, a breakthrough study held that addition of formaldehyde during the biomass preatment produces a soluble fraction of lignin which upon subsequent hydrogenolysis yields nearly theoretical amount of guaiacyl and syringyl monomers (47 mol % of Klason lignin for beech and 78 mol % for a high-syringyl transgenic poplar). The amount of these monomers produced was three to seven times more than that obtained without formaldehyde (Shuai et al., 2016). Later, it was found that aldehydes decondense the otherwise condensed linin macromolecules and stabilizes them in uncondensed form (Amiri et al., 2019). This form of lignin following subsequent hyrogenolysis produces highly selective monomers.

The three-dimensional folded structure of lignin impedes its hydrogenolysis into monomeric compounds. In an attempt to overcome this hurdle, the processes of oxidation and hydrogenolysis were combined. The Kraft lignin was treated with hydrogen peroxide which successfully broke the intramolecular hydrogen bonds and stretched its three-dimensional folded geometry in alkaline aqueous medium (Qi et al., 2017). The stretched lignin molecules were subjected to catalytic hydrogenolysis in the presence of Ni catalyst supported by the ZSM-5 zeolite. Due to prevention of repolymerization/self-condensation and higher monomeric yield, 83 wt% lignin was converted from Kraft lignin into oil (Qi et al., 2017).

As we know, the formation of selective delignification products depends upon various factors, besides the extraction process being employed. During these processes, diverse bonds that could otherwise lead to particular products are broken/modified. However, a group of reaserchers interestingly stabilized α, γ-diol group of lignin by the addition of acetaldehyde and propionaldehyde to prevent its condensation while extraction (Lan et al., 2018). The subsequent hydrogenolysis of the α, γ-diol-stabilized lignin in the presence of Pd/C catalyst based on Klason lignin (48% from birch, 20% from spruce, 70% from high-syringyl transgenic poplar) yielded 80% of 4-n-propanolsyringol monomer with high selectivity. Besides, the hydrogenation of this protected lignin using Ni/C as catalyst also yielded impressive selectivity (78%) of the same product (Lan et al., 2018). Therefore, protection of specific lignin linkages could prove more advantageous to depolymerize lignin into selective monomers, even after diversifying the downstream steps/catalysts employed.

#### Solvolysis

Lysis of a substance in the presence of a solvent is called solvolysis. Solvolysis of lignin or lignocellulosic biomass is one of the diverse fractionation methods which uses aqueous (hydrolysis) or non-aqueous (methanol, ethanol, acetone, etc.) solvents to break the stable ether, ester, C-C, and other bonds in the lignin biopolymer. Aqueous solvolysis may use sub or super critical water at relatively milder conditions to break lignin. It has many advantages, like, miscibility in a number of organic and inorganic compounds, thermal stability, low viscosity, and high diffusivity (Abad-Fernández et al., 2020). Incidences of rapid delignification were reported using supercritical water oxidation (Perez et al., 2019). Sometimes solvolysis is coupled with hydrogenolysis for the selective synthesis of aromatic compounds (Cheng et al., 2018). However, the main drawback lies in the re-polymerization of degradation products to form oligomers/larger fragments. Many researchers introduced phenols, organic solvents, bases, homo- and heterogeneous catalysts to avoid the re-polymerization/char formation (Yuan et al., 2010; Roberts et al., 2011; Mahmood et al., 2013; de Carvalho and Colodette, 2017).

While performing acid solvolysis of lignin, formation of monomers/desired products depends upon the reaction temperature and reaction time, apart from the source of lignin. When lignocellulose from hardwood, softwood, and grasses was pre-treated with dilute sulphuric acid to check the time and temperature dependency of product formation, xylose and mannose marked the initial intermediary products (Świątek et al., 2020). Regardless of the source of biomass, temperature elevation positively affected the formation of furfurals and organic acids. However, large amount of formic acid was obtained from the grass biomass (Świątek et al., 2020). Therefore, it can be concluded that acids or other additives can help in preventing the re-polymerization, however, temperature elevation will always play a major role in the process. Besides, this process also requires higher acidic concentrations which led to the formation of toxic compounds, such as, furfural and hydroxymethylfurfural (HMF) (Monlau et al., 2014).

On the other hand, alkali pre-treatment of lignocellulose is considered to be a promising alternative as it can process at low temperature and pressure conditions. Typically, NaOH and KOH are used which breaks the lignin-carbohydrate bonds (Xu et al., 2021). The biggest hindrance in this method is the larger retention time (hours or days). As far as the efficiency and economic conditions are concerned, calcium hydroxide and sodium hydroxide are the most suitable bases used (Shah et al., 2022). Although alkali pre-treatment is comparatively more effective, the usage of alkalis in larger amounts led to the corrosion of reactors and may contribute to chemical pollution as well.

As far as the non-aqueous solvolysis is concerned, ethanol is regarded as the most efficient solvent. Apart from acting as a hydrogen-donor, it functions as a capping agent to stabilize the C- and O-alkylation in the highly reactive phenolic intermediates, thereby preventing their re-polymerization into oligomers (Kim and Kim, 2018). Supercritical ethanol was found to be significantly more effective in producing monomers and avoiding char than supercritical methanol (Huang et al., 2015). Unfortunately, char formation was found to occur in many of the reactions. For instance, upon treatment of organosolv lignin with ethanol at sub and supercritical temperatures (200°C, 275°C, and 350°C), char and oil production accompanied the phenolics (Kim et al., 2013). To prevent the char formation in ethanol solvolysis, a copper-containing mixed oxide was added to the supercritical ethanol (Huang et al., 2014). Similarly, Güvenatam et al. (2016) used metal acetates, metal chlorides, and metal trifluoromethanesulfonates as Lewis acid catalysts for the deconstruction of soda lignin at 400°C in supercritical ethanol and water. Supercritical methanolic depolymerization of lignin also yielded better results when coupled with hydrodeoxygenation/reduction in the presence of metal catalysts. In a comparative degradation analysis of maple wood and lignin extracted from it in supercritical methanol followed by hydrodeoxygenation in the presence of copper metal catalysts, the initial products formed included phenols (P or H), guaiacols (G), and syringols (S) with deoxygenated 1 to 3-carbon (C_1_–C_3_) alkyl tails (McClelland et al., 2019). The amount of repolymerization was observed to be more in the degradation products of maple wood due to the higher amount of lignin. Thus, supercritical fluids are capable of pre-treating lignocellulose at relatively mild conditions, with higher monomeric production and lower usage of chemicals and lesser yield of fermentation inhibitors, and more susceptibility to enzymatic hydrolysis (Escobar et al., 2020).

### Biological Delignification

In nature, lignin is degraded by an array of micro-organisms. Separation of lignin from the lignocellulosic biomass followed by depolymerization and mineralization is accomplished through synergistic interactions between bacteria and fungi, the latter being the major key players (Lee et al., 2019; Atiwesh et al., 2021). Typically, brown rot, white rot, and soft rot fungi are regarded as the main mycobionts involved in delignification, while white rot fungi being more effective. Few white rot fungi, apart from delignification, are able to degrade other components of lignocellulosic biomass as well, while others are selective delignifiers (Janusz et al., 2017). For example, *Heterobasidium annosum* (Maijala et al., 2003), *Phlebia* spp. (Arora and Sharma, 2009), *Physisporinus rivulosus* (Hilden et al., 2007), *Irpex lacteus* (Duan et al., 2018), *Dichomitus squalens* (Marinović et al., 2018), *Phanerochaete chrysosporium*, *Pleurotus ostreatus* (Kerem et al., 1992), *Ceriporiopsis subvermispora* (Honda et al., 2019), and *Trametes versicolor* (Bari et al., 2019). In comparison to fungal studies, research on potential of bacterial lignin deconstruction is scarce. Some of the lignin degrading bacteria mostly belong to the actinomycetes, firmicutes, and proteobacteria, mainly encompassing *Sterptomyces*, *Rhodococcus*, *Pseudomonas*, and *Bacillus* strains (Bugg et al., 2011; Wilhelm et al., 2019).

Fungal assemblages have been observed to depolymerize the lignin into monomers/low-molecular weight aromatics which are subsequently assimilated by bacteria for carbon and energy (Bugg and Winfield, 1998). Although bacteria are reported to play little role in direct delignification, mineralization is predominantly governed by them (Kamimura et al., 2017). Recently, bacterial systems have gained essence in lignin valorization due to their inherent processes, which are capable of channelizing multiple aromatic streams into a uniform compounds (Linger et al., 2014). Therefore, challenges associated with lignin heterogeneity and undesirable outcome can potentially be overcomed. Moreover, enzymatic hydrolysis yields monomers and subsequently mineralize them which prevents the re-polymerization of products. For example, in a bioconversion kinetic study, *Pseudomonas putida* KT2440 was incubated with two types of lignins—Kraft lignin and synthetic dehydrogenopolymer (DHP), and the phenolic monomers (dihydroferulic acid) formed transitorily were metabolized within 24 h (Rouches et al., 2021). Lignin degrading ability of such microbes are attributed to their potential to produce extracellular enzymes, called “ligninases”, which mainly include laccases, peroxidases, oxidoreductases, dye-decolorizing peroxidases, and versatile peroxidases.

Laccases (*benzenediol:oxygen oxidoreductases*, EC 1.10.3.2) are N-glycosylated extracellular multi-copper oxidases containing histidine-rich copper-binding domains (Messerschmidt and Huber, 1990). To date, laccases are reported from plants, fungi, and bacteria, however, most of the laccases characterized pertaining to their delignification ability are of fungal origin (Chauhan et al., 2017), with scarce incidences in bacteria. Although fungal laccases are commonly used owing to their higher redox potential, prokaryotic laccases which exhibit extreme thermostability, pH stability, and rapid proliferation make them ideal for industrial applications (Janusz et al., 2020). Unlike peroxidases, laccases do not produce toxic peroxide intermediates and function without the cofactors, like, NAD(P)H (Santhanam et al., 2011). Due to comparative low redox potential than other ligninases, laccases act only upon the phenolic compounds, however, their activity can be extended to non-phenolic compounds as well by the addition of mediators, such as, vanillin, p-coumaric acid, acetosyringone, syringaldehyde, 3-hydroxyanthranilic acid (HAA), 1-hydroxybenzotriazole (HBT), 2,2′-azino-di(3-ethylbenzthiazoline-6-sulfonic acid) (ABTS) (Kontro et al., 2020). Apart from enhancing the substrate specificity and oxidative capacity of laccases, mediators also prevent re-polymerization (Grelier and Koumbe, 2016; Huber et al., 2016). Together with mediators, these enzymes are able to perform Cα-Cβ, β ether and Cα oxidative cleavage. Besides mediators, some other enzymes are also used to enhance the lignin degradation by enzymatic hydrolysis viz., aryl-alcohol oxidase, catechol 2, 3-dioxygenase, feruloyl esterase, lipases, and quinone reductases (Kumar and Chandra, 2020).

Lignin peroxidases *(1,2-bis(3,4-dimethoxyphenyl)propane-l,3-diol:hydrogen-peroxide oxidoreductases,* EC 1.11.1.14) (LiP) are a group of heme-containing glycoproteins which use hydrogen peroxide (H_2_O_2_) for their activity to oxidize both phenolic and non-phenolic lignin constituents (Chowdhary et al., 2019). They exhibit high redox potential, less substrate specificity, besides maintaining their activity at low pH (Falade et al., 2017). These peroxidases preferably oxidize methoxylated aromatic ring without a free phenolic group. LiPs are used commercially to mineralize a variety of recalcitrant aromatic compounds, like, three- and four-ring polyaromatic hydrocarbons, polychlorinated biphenyls, and natural dyes (Falade et al., 2017). They mostly work by transforming the lignin degradation fragments released by the activity of manganese peroxidase.

Manganese peroxidases [Mn (ll): hydrogen-peroxide oxidoreductases, EC 1.11.1.13] (MnP) are glycosylated, heme-containing extracellular enzymes which require Mn^2+^ to oxidize lignin (Xu et al., 2017). On account of the lower redox potential of MnP-Mn complex in comparison to lignin peroxidases, it can oxidize both phenolic and non-phenolic substrates via lipid peroxidation reaction (de Eugenio et al., 2021). They are produced by the majority of fungi and acts by oxidising Mn^2+^ to Mn^3+^ which further oxidizes the phenolics into pheoxy radicals. These radicals, upon further reaction can result in the release of CO_2_ or cleave the alkyl-alkyl or/and alkyl-phenyl bonds to form monomers/low molecular weight intermediates, such as, quinones and hydroxyl quinines (Kumar and Chandra, 2020).

Dye decolourizing peroxidases (*Reactive-Blue-5: hydrogen-peroxide oxidoreductase,* (EC 1.11.1.19) include those peroxidases (DyP) which decolorize anthraquinone derived dyes. The ability of delignification in such enzymes is due to the structural similarity of these dyes with that of lignin (Catucci et al., 2020). Therefore, these microbes can be detected through their dye-decolourizing capability. Two types of dye decolourizing peroxidases- DypA and DypB were identified based on bioinformatics analysis. DypB was characterized from a soil bacterium *Rhodococcus jostii* RHA1 which was able to degrade the Kraft lignin via C(α)-C(β) bond cleavage (Ahmad et al., 2011). Similarly, BaDyP, a dye decolourizing peroxidase from *Bacillus amyloliquefaciens* was found to decolourize dyes and degrade guaiacylglycerol-β-guaiacyl ether (GGE), a phenolic β-ether lignin model (Yang et al., 2019).

Versatile peroxidase (VP) *reactive-black-5:hydrogen-peroxide oxidoreductase*, EC 1.11.1.16) are the monomeric glycoproteins (40–50 kDa) with two conserved calcium binding sites and four conserved disulfide bridges, and are capable of acting without redox mediators (Perez-Boada et al., 2005). These constitute a unique type of lignin oxidizing peroxidases which possess the combined catalytic potential of manganese peroxidases and lignin peroxidases, the property that makes them the hybrid enzymes. However, direct cleavage of high reduction potential substrates and independent oxidation of Mn^2+^ ions makes them different from LiP and MnP (Camarero et al., 1999). Also, unlike the other oxidases, they work without the redox mediators (Moreira et al., 2007).

Delignifiers are widely distributed in nature and can be found in soil, water, air, coal, gut of rumens and termites, and inside the plants as endophytes (Xiong et al., 2014; Suman et al., 2016; Wang et al., 2016; Li et al., 2017; Zhou et al., 2017).

### Endophytes as Potent Delignifiers

**FIGURE 3:**
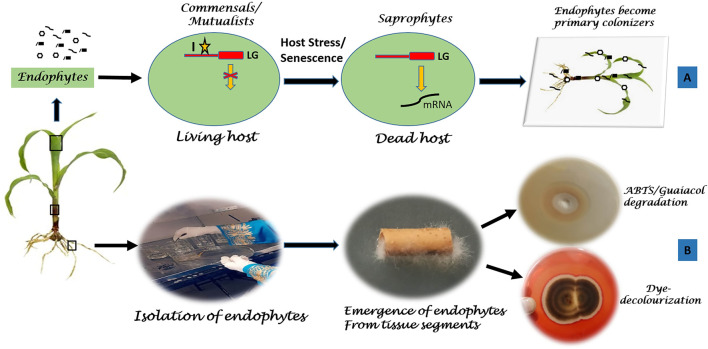
Endophytes as lignin decomposers. **(A)** Schematic overview of natural endophytic delignification. **(B)** Pictorial view of *in vitro* investigation of endophytic delignification. Where I, Inhibitor; LG, ligninase gene; ABTS, [2, 2′-azino-bis (3-ethylbenzothiazoline-6-sulfonic acid].

Endophytes are regarded as the fascinating microbes due to their asymptomatic existence inside the plants assisted by numerous secondary metabolites and enzymes. Most of the extracellular enzymes produced are being correlated to their penetration/entry inside the host plant (Abdalla et al., 2020). However, the interesting fact lies in their stable colonization *in planta*, where genes coding for these enzymes might be temporarily shut off, by the time any kind of stress prevails or host dies (Promputtha et al., 2007; Nelson et al., 2020). After host’s death, as a survival strategy, many endophytes switch their lifestyle to saprophytism and invade as the primary colonizers to feed on the dead host, thereby initiating decomposition of wood (lignocellulose) via the production of various enzymes (Song et al., 2017) (Figure 3A). Certain endophytes may join the degradation process after sometime but function upto the last stage of decomposition (Promputtha et al., 2010). This capability makes them competent for degrading numerous recalcitrant aromatic compounds including lignin into monomers, ultimately adding basic nutrients to soil. Hence, endophytes play an important role in bio-geochemical cycling.

Exploration of endophytic microbes as agents of delignification is an emerging field. *Monotospora* sp. was the first endophytic fungus isolated from *Cynodon dactylon* that was reported to produce laccase (Wang et al., 2006). Most of the studies have focussed on the isolation of endophytes from healthy plant tissues followed by their investigation for ligninase producing ability or degradation of certain lignin-based substrates (Figure 3B). Both endophytic bacteria and fungi are known to produce ligninases in artificial cultures with ABTS (2, 2′-azino-bis (3-ethylbenzothiazoline-6-sulfonic acid) and guaiacol (2-Methoxyphenol) as the frequently used substrates (Table 2). For example, endophytic fungi *Pringsheimia smilacis*, *Neofusicoccum luteum*, *Neofusicoccum austral*, *Hormonema* sp., and *Ulocladium* sp., isolated from the xylem of *Eucalyptus* spp. were found to degrade lignin-based model compound ABTS, the potency was associated with laccase production (Fillat et al., 2016). Similarly, *Colletotrichum gloeosporioides*, an endophyte of *Piper betle* produced laccase after growing in the medium containing Guaiacol (Sidhu et al., 2014). *Trichoderma asperelloides* LBKURCC2, a palm endophyte degraded both ABTS and guaiacol (Pisacha et al., 2020). Eight endophytic fungi isolated from *Brunfelsia uniflora* showed laccase activity (Marsola et al., 2022). Furthermore, five dark septate endophytic fungi derived from roots of *P. merkusii* also showed lignolytic activity (Akhir et al., 2022). Recently, it was observed that fungal endophytes of the grass species, *Festuca sinensis*, *Stipa purpurea*, and *Achnatherum inebrians* plays an important role in the litter decomposition of their hosts and nutrient trasition of ecosystem (Song et al., 2022).

**TABLE 2:** Lignin degrading ability shown by endophytes on various substrates.

Endophytes	Source	Substrate	References
*Xylaria* sp.	*Abies alba*	0.5% Indulin A 0.025% Polyfon H	Carroll and Petrini, (1983)
*Fusarium proliferatum* NRRL 31071	Wheat	ABTS	Anderson et al. (2005)
*Monotospora* sp.	*Cynodon dactylon*	ABTS	Wang et al. (2006)
*Chaetomium globosum*	*Glinus lotoitks*	ABTS	El-Zayat, (2008)
*Alternaria alternata* *A. solani* *Gonatorrhodiella parasitica* *Monascus ruber* *Trematosphaeria mangrovei* *Ulocladium*	*Paduia* sp. *Pterocladia* sp. *Cystoseira* sp. *Sargassum* sp. *Corallina* sp. *Ulva* sp*.*	Guaiacol	Atalla et al. (2010)
*Coccomyces sinensis*	*Camellia japonica*	Decaying leaves	Hirose et al. (2013)
P6MT1 and P2MT1	*Eucalyptus globulus*	Guaiacol 1-napthol	Sathish et al. (2012)
*Chaetomium* sp. *Colletotrichum* sp. *Aspergillus niger* *Fusicoccum* sp. *Discosia* sp. *Xylaria* sp. *Phoma* sp. *Isaria* sp. *Pestalotiopsis disseminata Penicillium* sp.*Colletotrichum truncatum*	*Alpinia calcarata* *Bixa orellana* *Calophyllum inophyllum Catharanthus roseus*	1-napthol	Sunitha et al. (2013)
*Colletotrichum gloeosporioides*	*Piper betle*	Guaiacol	Sidhu et al. (2014)
*Pantoea ananatis* Sd-1	Rice seeds	ABTS Guaiacol	Shi et al. (2015)
*Daldinia* sp.	*Cupresus torulosa*	Guaiacol Nephthol ABTS Syringaldehyde	Agrawal and Chanyal, (2017)
*Hormonema* sp. *Pringsheimia smilacis* *Ulocladium* sp. *Neofusicoccum luteum, Neofusicoccum australe*	*Eucalyptus*	ABTS	Fillat et al. (2016)
*Myrothecium verrucaria*	*Cajanus cajan*	ABTS	Sun et al. (2017)
*Bacillus* sp. *Klebsiella* sp. *Pseudomonas* sp. *Clavobacter* sp. *Micrococcus* sp. *Xanthomonas* sp. *Enterobacter* sp. *Serratia* sp. *Escherichia coli*	*Musa acuminata* *Hevea Brasiliensis*	Guaiacol Tannic acid	Wins et al. (2019)
*Trichoderma asperelloides* LBKURCC2	Palm	ABTS Guaiacol	Pisacha et al. (2020)
*Bartalinia pondoensis* *Epicoccum sorghinum* *Cladosporium* sp. *Truncospora tephropora* *Mucor circinelloides*	*Ecklonia radiata*	ABTS	Perkins et al. (2021)
*Chaetomium globosum*	*Hibiscus manihot*	Larch sawdust	Dou et al. (2022)

On the other hand, as discussed earlier, reports on delignifying endophytic bacteria are comparatively lagging. An endophytic bacterium, *Pantoea* sp. Sd-1 isolated from the rice seeds however, was found to degrade both ABTS and guaiacol (Shi et al., 2015). Bacterial endophytes viz., *Bacillus* sp*., Klebsiella* sp., *Pseudomonas* sp*.*, *Clavobacter* sp*.*, *Micrococcus* sp., *Xanthomonas* sp., *Enterobacter* sp., *Serratia* sp., and *Escherichia coli* associated with *Musa acuminata* and *Hevea brasiliensis* also showed the ability to metabolize guaiacol (Wins et al., 2019). As reported, microbial degradation of ABTS is mainly associated with the oxidative catalysis by laccase, however, guaiacol is oxidized to catechol by cytochrome P450 monooxygenases (García-Hidalgo et al., 2019).

Certain endophytes are capable of producing multiple lignin degrading enzymes. For instance, marine fungi *Alternaria alternata*, *A. solani*, *Gonatorrhodiella parasitica*, *Monascus ruber*, *Trematosphaeria mangrovei*, and *Ulocladium* isolated from *Paduia* sp. *Pterocladia* sp. *Cystoseira* sp. *Sargassum* sp., and *Corallina* sp. *Ulva* exhibited laccase (Lac), lignin-peroxidase (LiP) and manganese-dependent peroxidase (MnP) activities (Atalla et al., 2010). Similarly, *Myrothecium verrucaria* produced all the above-mentioned ligninases (Su et al., 2018). The laccase procured also displayed various degrees of decolourization of several dyes in the presence of ABTS redox mediator. An endophytic fungus *Fusarium proliferatum* strain NRRL 31071 isolated from wheat showed extracellular laccase and aryl alcohol oxidase activities (Anderson et al., 2005). Likewise, *Fusarium sambucinum* from a mangrove plant *Cananeia* sp. showed lignin peroxidase and manganese peroxidase activity (Martinho et al., 2019). Also, basidiomycetous fungi endophytic to a macroalga *Ecklonia radiata* produced laccase and lignin peroxidase under oxic conditons (Perkins et al., 2021). Recently, *Colletotrichum acutatum*, *Nigrospora sphaerica*, *Exserohilum rostratum*, *Diaporthe phaseolorum*, and *Pestalotiopsis arceuthobii* isolated as endophytes from *Taxillus chinensis* showed manganese peroxidase and lignin peroxidase activity (Song et al., 2022). Also, an endophytic fungus *Diaporthe phaeolorum* recovered from the leaves of *Dillenia indica* exhibited Lac, LiP, and MnP activities (Kumar and Prasher, 2021). Upon investigating the effect of various carbon and nitrogen sources on the growth and production of ligninolytic enzymes, maximum laccase (44.5 U/ml), lignin peroxidase (3.5 U/ml), and manganese (6.88 U/ml) activity was found in pectin, sucrose, and glucose, respectively. Therefore, such endophytes can simultaneously break diverse bonds in lignin biopolymer and release different depolymerization products. As far as physical/chemical depolymerisation is concerned, different conditions needs to be maintained (high temperature, pH, irradiation), and many chemicals are to be supplemented in order to breakdown multiple kinds of linkages. Therefore, employing an endophyte with multiple ligninase ability can perform this function in an eco-friendly way with less energy consumption.

Being comparatively unexplored for delignification ability, endophytes can act as the promising tools for unearthing novel ligninases. As for example, an endophytic bacterium *Pantoea* sp. Sd-1 isolated from rice seeds showed outstanding ability of degrading lignin and rice straw (Xiong et al., 2014). This effect increased upon addition of glucose and 0·5% peptone to the rice straw or Kraft lignin containing medium. Upon genomic analysis of Sd-1, four putative laccases—Lac1, Lac2, Lac 3, and Lac4 were identified (Shi et al., 2015). However, only Lac4 possessed the complete signature sequence for laccase activity and resembles known bacterial multi-copper oxidases upto 64%. Apart from its acid-stable nature, recombinant Lac4 was capable of oxidizing both the phenolic and non-phenolic compounds, decolourize numerous synthetic dyes, and degrade ABTS and guaiacol at acidic pH (Shi et al., 2015). Similarly, a novel laccase enzyme, *Colletotrichum gloeosporioides* gr. laccase was recovered from an endophytic fungus, *Colletotrichum gloeosporioides* colonizing *Piper betle* (Sidhu et al., 2014). Also, a novel endophytic laccase (LACB3) isolated from *Phomopsis liquidambari* was cloned and investigated for its potential to promote peanut growth. Upon application in soil, laccase enhanced peanut biomass by 12% while downgraded the respective contents of coumaric acid, vanillic acid, and 4-hydroxybenzoic acid by 21%, 27%, and 40% (Wang et al., 2014). Recently, an extensive exploration revealed a novel laccase producing endophytic fungus *Chaetomium globosum* from *Hibiscus manihot* L. which selectively degraded lignin in larch sawdust into p-Hydroxyphenyl and Guaiacyl units (Dou et al., 2022). This indicates that discovery of novel microbial strains with the recognition of their enzyme systems for lignin degradation is imperative for the effective conversion of lignin into fuel and chemicals.

Despite being an eco-friendly, economical, and sustainable method of delignification, the slower rate of reaction halts the applicability of enzymatic lignin depolymerization at industrial scale. Although, white and brown rot fungi (Basidiomycetes) are regarded as the most influential and industrially competent enzymatic delignifiers, endophytic fungi showed more efficiency in saccharification of wood (and delignification) compared to that of *Trametes* sp. I-62, a white rot fungus, as exemplified in *Eucalyptus* (Fillat et al., 2016). It indicates that endophytes can surpass the enzymatic potential of basidiomycetes in depolymerizing lignin. This ability of certain endophytes, if not all, may be accompanied by their existence as primary colonizers or their competition on the decaying host with the potent basidiomycetes. Hence, explorations and extensive studies to unravel the delignifying endophytes are pivotal to unearth the more effective lignin degraders which can overcome the limitations of microbial delignification to be employed industrially.

### Endophytic Dye-Decolourization: An Activity in Correlation With Lignin Deconstruction

On account of slow degradability and frequent binding property of lignocellulose to cationic molecules, complex chelating compounds get accumulated in the environment (Chandra et al., 2012). Therefore, industrial waste water from pulp industry potentially contributes to the environmental pollution (Sadh et al., 2018; Singh and Chandra, 2019). Several endophytic explorations are being made with respect to their dye-decolourizing potential, which find their applications in bioremediation and waste water pre-treatment. Interestingly, this ability is in positive correlation with their delignification potential owing to the structural similarity of aromatic dyes to that of lignin biopolymer. Various endophytes found to decolourize aromatic dyes and the enzymes involved are mentioned in Table 3. Although diverse methods are used in degradation of textile effluent dyes, endophytes can essentially augment the biodegradation in a sustainable and eco-friendly manner (Goud et al., 2020). A novel bacterial endophyte *Exiguibacterium profundum* strain N4 was isolated from a plant, *Amaranthus spinosus* growing on a textile dye effluents-contaminated site in Rajasthan, India (Shilpa and Shikha, 2015). It efficiently decolourized 901 ppm of Reactive Black 5 (RB5), an azo dye upto 84.78% after incubation at 30°C for 12 h. HPLC, GC-MS, and UV-Vis spectroscopy analysis confirmed biodegradation of the dye. Addition of glucose (10 g/L) and beef extract (2 g/L) significantly enhanced the decolourizing ability of the endophyte. Interestingly, another bacterium *Proteus mirabilis* isolated from the root nodules of Cactus decolourized and degraded RB5 under static conditions (Bibi et al., 2020). Upon elevation of temperature to 37°C and incubation time to 72 h, the activity was maximized upto 90%.

**TABLE 3:** Endophytes capable of degrading and decolorizing aromatic dyes.

Endophyte	Source	Dye-decolourized	Enzyme produced	References
CMUX144	Native plants of Doi Suthep-Pui National Park, Thialand	Poly R-478	Manganese independent peroxidase	Urairuj et al. (2003)
Sterile mycelium (S11309)	*Brucea javanica*	Poly R Agar	_	Choi et al. (2005)
*Corynespora cassiicola*	*Magnolia liliifera*	Poly R	_	Promputtha et al. (2010)
P3ML1, P6MT1, P5MT1 and P2MT1	*Eucalyptus globulus* and *Eucalyptus citriodora*	1-naphthol	_	Satish et al. (2012)
AEF17, AEF19, AEF22 and AEF25	*Canavalia rosea*, *Ipomea pescaprae* and *Spinifex* sp.	M2R (BM2R), Black-B (BB) and Orange M2R(OM2R), Yellow MR(YMR), Red BSID (RBSID), Manenta MP (MMP), Blue MR (BMR), Orange 3R (O3R) and Brown GR (BGR)	_	Muthezhilan et al. (2014)
*Exiguobacterium profundum* N4	*Amaranthus spinosus*	Reactive black	_	Shilpa and Shikha, (2015)
*Pantoea ananatis* Sd-1	Rice seeds	Remazol Brilliant Blue R	Laccase (Lac4)	Shi et al. (2015)
*Marasmius cladophyllus* UMAS MS8	*Melastoma malabathricum*	RBBR	Laccase	Sing et al. (2017)
*Cladosporium cladosporioides*	*Ipomoea hederifolia*	Navy Blue HE2R (NB-HE2R)	Lignin peroxidase, Tyrosinase, Laccase	Patil et al. (2016)
*Bacillus* *Microbacterium* *Halomonas*	*Typha domingensis*, *Pistia stratiotes*, and *Eichhornia crassipes*	Textile effluents	_	Shehzadi et al. (2016)
*Diaporthe* sp.	*Portulaca* weed	crystal violet, methyl violet, malachite green, and cotton blue	_	Ting et al. (2016)
*Daldinia* sp. KCT34	*Cupressus torulosa*	Congo red, Rosebangal, Orange G, and Rhodamine B	Laccase	Agrawal and Chanyal (2017)
*Phlebia* sp. *Paecilomyces formosus*	*Helianthus annuus*	Reactive Blue 19 and Reactive Black 5	Laccase	Bulla et al. (2017)
*Pestalotiopsis versicolor*	*Cupressus torulosa*	Congo red, Rhodamine B, and Orange G	_	Rajput et al. (2017)
*Myrothecium verrucaria*	*Cajanus cajan*	Congo red, Methyl orange, Methyl red, and Crystal violet	Laccase	Sun et al. (2017)
*Phomopsis* sp.	*Simarouba glauca*	RBBR	Laccase	Navada et al. (2018)
*Bjerkandera adusta* SWUSI4	*Sinosenecio oldhamianus*	Cotton blue (CB), crystal violet (CV), malachite green (MG), and methyl violet (MV)	Manganese peroxidase and lignin peroxidase	Gao et al. (2020)
*Colletotrichum gloeosporioides*	*Salacia chinensis*	Methylene blue and congo red	_	Sheik et al. (2020)
*Phlebia acerina* MY51	*Vaccinium ashei*	Reactive red, reactive black	Lignin peroxidase, laccase, and manganese peroxidase	Zhang and Hou, (2020)
*Penicillium megasporum*	*Cupressus torulosa*	Congo red, Orange G, and Rhodamine B	Laccase	Agrawal et al. (2021)
*Bartalinia pondoensis* *Epicoccum sorghinum* *Cladosporium* sp. *Truncospora tephropora* *Mucor circinelloides*	*Ecklonia radiata*	RBBR	Manganese peroxidase and sulfur-containing lignin peroxidase	Perkins et al. (2021)
*Penicillium oxalicum*	*Tecomella undulata*	Methylene blue	_	Mathur et al. (2021)
CPP and KSP	*_*	Congo red	Laccase and tyrosinase	Melati et al. (2021)
*Pseudopestalotiopsis theae* *Astrocystis bambusae*	*Usnea* sp. (Lichen)	Malachite Green	_	Ting et al. (2016)
*Fusarium proliferatum*	*Cymbopogon citratus*	Methyl Violet, followed by Crystal Violet (23.8%) and Malachite Green	_	Schuster and Ting (2021)
*Aspergillus niger* *Syncephalastrum racemosum* *Penicillium citrinum* *Sterile mycelia*	*Avicennia marina*	Congo red, Methylene blue, Malachite green, and Rhodamine B	Laccase, Lignin peroxidase, Manganese peroxidase	Obanan et al. (2022)

Dye decolourization by endophytes pertains to their potential of synthesizing laccases and peroxidases. An endophytic fungus M*arasmius cladophyllus* isolated from senduduk plant, *Melastoma malabathricum* was initially found to decolourize synthetic dyes, such as, Rubidium bromide (RBBR) (Ngieng et al., 2013). Upon detailed analysis, decolourizing ability was found to be associated with biodegradation of these dyes via the production of laccase and lignin peroxidase enzymes in the medium containing RBBR (Sing et al., 2017). Dye decolourization in the subsequent generations was also faster even without the addition of a mediator. Similarly, a dye decolourizing laccase was produced by a novel endophyte My*rothecium verrucaria* MD-R-16 colonizing *Cajanus cajan* (Sun et al., 2017). The laccase decolourized dyes, such as, Congo red, Methyl orange, Methyl red, and Crystal violet in the presence of ABTS as mediator. Apart from its higher production, laccase was found to be acid-stable and thermostable upto 55°C. As stated by Davis and Moon (2020), lignin valorization will be facilitated by novel microbes capable of utilizing lignin or allied aromatic compounds. Therefore, endophytes can act as the promising tools for exploring potent ligninases possessing the ability of degrading lignin and other waste products, thereby valorizing lignin and performing phytoremediation, simultaneously. In a recent research, fungal dye-decolourizing peroxidases (DyPs) were studied for their gene sequences. Upon phylogenetic analysis of the DyPs gene sequences available in public domains, seven fungal clades were distinguished on the basis of sequence similarity network (Adamo et al., 2022). Sequences pertaining to one of the clades showed divergence from others having highest number of *N*-glycosylation sites, N-terminal sequence peptides for secretion, lower isoelectric points and hydropathy indices (Adamo et al., 2022). Interestingly, the putative proteins from the distinct clade were lacking from brown rot and ectomycorrhizal fungi which have lost the ability of enzymatic delignification. This study did not highlight whether the distinct clade occupied endophytic fungi nor did it identify these fungi in other clades. However, it showed the diversification of fungi based on DyPs genes. Hence, in-depth explorations regarding endophytic genes encoding dye-decolourizing enzymes are required to unearth the potential genes alongwith their properties and variations which could further be used in improving the enzyme secretion of these microbes using modern techniques.

### Endophytes: A Promising Source of Stable Ligninases

To be compatible with industrial applications, stability or tolerance of enzymes to varied temperatures, pH, and high adaptability is the requisite. Extremophilic potential of ligninolytic enzymes is regulated by diverse factors, such as, specific genes, hydrophobic interactions, ion pairs, disulfide bridges, salt bridges, and hydrogen bonding between amino acids (Pace et al., 2014). Endophytes are well known to produce thermotolerant and acido-tolerant enzymes. For instance, endophytic *Periconia* sp. BCC2871 was found to produce thermotolerant β-glucosidase, BGL I (Harnpicharnchai et al., 2009). After cloning the complete gene encoding BGL1 into *Pichia pastoris* KM71, the recombinant enzyme exhibited similar characteristics to that of its native counterpart. Besides showing optimum activity at 70°C with pH of 5–6, engineered BGL1 retained its activity after several cycles of incubation. More so, it efficiently degraded rice straw into simple sugars indicating its potential for application in biomass conversion. Likewise, an acidotolerant and thermotolerant laccase was produced by endophytic *Phomopsis liquidambari* (Wang et al., 2014). The subsequently cloned and expressed enzyme showed remarkable features to be utilized at industrial scale. A novel acid-stable laccase Lac4 identified from the genome of an endophytic bacterium *Pantoea ananatis* Sd-1 was found to degrade lignin and decolourize various dyes (Shi et al., 2015). The enzyme was able to act on both phenolic and non-phenolic compounds under acidic conditions (pH 2.7–4.5) at a temperature range of 30°C–50°C. Same featured laccase was produced by another novel fungus *Myrothecium verrucaria* MD-R-16 endophytic to *Cajanus cajan* (Sun et al., 2017). The optimal conditions for laccase production included incubation at 30°C and pH 6.22 for 5 days, however, the enzyme also showed relative stability at pH 4.5–6.5 and temperature range of 35°C–55°C.

Although endophytes from all kinds of habitats can be exploited for their potency to synthesize stable enzymes, those from extreme environments have attracted attention due to their ability to produce higher amounts of valuable metabolites and enzymes (Singh and Dubey, 2018). This ability is attributed to the higher exposures to diverse array of stress factors, such as, heat, cold, salinity, high UV radiations, and numerous other oligotrophic conditions. All the organisms well adapted to such hostile environments have been observed to synthesize a plethora of stable enzymes (Sarmiento et al., 2015; Monsalves et al., 2020). However, of these microbes, endophytes being primary colonizers on their dead hosts could be the potent initiators of delignification. Similar to the endophytes recovered from contaminated sites which produce contamination tolerant enzymes (Lumactud et al., 2016; Goud et al., 2020), harsh habitats are the best sites to explore heat-stable, cold-stable, and pH stable enzymes (Martínez et al., 2018).

Interestingly, ligninolytic microbes from extremophilic environments, conventional proteins were found in completely denatured state (Chandra et al., 2017). For instance, a cold-adapted bacterium *Paraburkholderia aromaticivorans* AR20-38 isolated from the soil of Alpine coniferous forest in Italy (altitude of 1,724–1,737 masl) efficiently degraded the lignin monomers at low temperature (Magesin et al., 2021). Similarly, many endophytes explored from such habitats exhibited potent lignin deconstruction properties. For instance, from mangrove (high salinity, low pH) Brazilian plants, 19 endophytic fungi exhibited ligninoytic activities (Marinho et al., 2019; Marinho et al., 2020). Out of these, *Fusarium sambucinum*, *Diaporthe* sp., *Fusarium* sp., and *Hypocrea lixii* showed exceptional laccase, lignin peroxidase, and managnsese peroxidase activities. Conclusively, endophytes colonizing the plants growing in harsh environmental conditions could be the formidable source of lignin depolymerizing enzymes, therefore, explorations are required to exploit their maximum potential.

### Mechanism of Enzymatic Delignification

**FIGURE 4:**
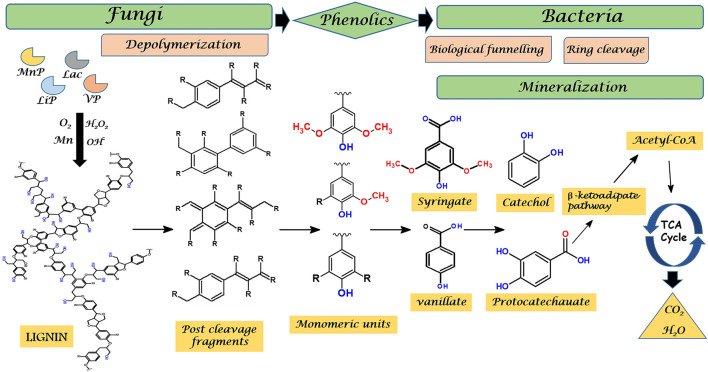
Pathways involving the role of fungi and bacteria in enzymatic degradation of recalcitrant lignin. Where Lac, Laccase; LiP, Lignin peroxidase; MnP, Manganese peroxidase; VP, Versatile peroxidase; TCA, Tricarboxylic acid.

Various microbes, including fungi and bacteria degrade lignin in nature which is accomplished by the complex synergy of diverse enzymes. Difficulty in delignification mainly occurs due to the presence of heterogeneous C-C linkages in phenylpropane building blocks (Castro-Sowinski et al., 2002). Fungal laccases and peroxidases are reported to oxidize lignin using oxygen (O_2_) and hydrogen peroxide (H_2_O_2_), respectively, which assist in the formation of free hydroxyl (OH¯) ions. These ions which are mainly produced by three pathways—cellobiose dehydrogenase catalyzed reactions, quinone redox cycling, and Fenton reactions catalyzed by glycopeptides (Dashtban et al., 2010), attack the plant cell wall components including lignin governing cleavage of various bonds. It has been observed that post cleavage fragments of lignin are more susceptible to attack by diverse lignin modifying enzymes (Kantharaj et al., 2017). Thus diverse fungi are more efficient in pre-treatment of lignin to their intermediates or low molecular weight heterogeneous compounds (5/6-carbon chains) (Kumar and Chandra, 2020). Intriguingly, several bacteria capable of delignification have evolved pathways to funnel these heterogeneous oligomers into common products through the process called biological funnelling. It occurs via upper pathways which functions as “biological funnels” for channelizing the lignin-derived heterogeneous aromatic compounds to common central intermediates, mainly protocatechuate or catechol (Eltis and Singh, 2018). Subsequently, these intermediates experience ring cleavage and are further converted into central carbon metabolism via β-ketoadipate pathway (Linger et al., 2014) (Figure 4).

Biological funnelling is the property that makes microbial delignification the most important method for valorizing heterogeneous lignin into common desired products. For example, demonstration on *Pseudomonas putida* KT2440 revealed its role in the conversion of both aromatic model compounds and heterogeneous lignin-derived streams into polyhydroxyalkanoates (mcl-PHAs) (Linger et al., 2014). These are the intracellular energy-rich, carbon storage compounds utilized in carbon-deficient conditions by numerous microorganisms. PHA’s find their applicability in pharmaceutical and biomedical industries, packaging materials, energy and chemicals (Tsang et al., 2019). Similarly, *Pseudomonas putida* KT2440 funnelled various lignin-derived species into *cis-cis* muconic acid which was subsequently hydrogenated to adipic acid, a dicarboxylic acid used in confectionery, fats, flavouring agents, and in the synthesis of nylon 6.6 (Vardon et al., 2015).

Interestingly, these microbes not only funnel the variable lignin based constituents into common products but the lignin from variable biomass sources also. For instance, an engineered bacterium *Novosphingobium aromaticivorans* DSM12444 modulated by a targeted gene deletion, used its native funnelling pathways for the conversion of guaiacyl (G), syringyl (S), and *p*-hydroxyphenyl (H) aromatic units into 2-pyrone-4,6-dicarboxylic acid (PDC), a powerful polyester precursor (Perez et al., 2018; Perez et al., 2019). Surprisingly, this microbe could also funnel the heterogeneous mixture of aromatic monomers formed by the chemical depolymerization of poplar lignin into PDC. Currently, biological funnelling of other heterogeneous and toxic compounds has also gained essence (Borchert et al., 2022).

### Endophytic Catalysis of Lignin to Value-Added Products

**FIGURE 5:**
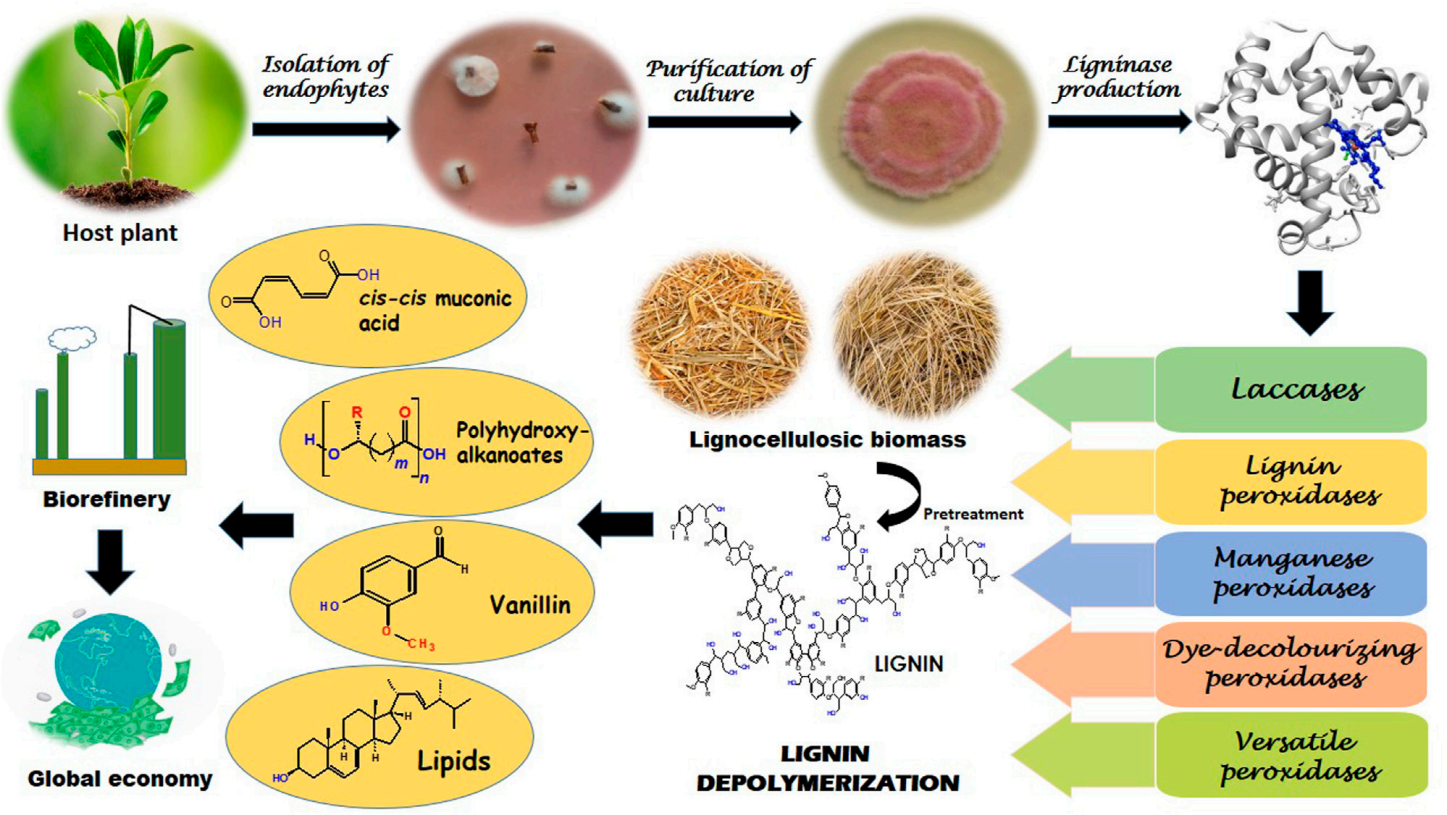
Schematic representation of the process involved in endophytic lignin valorization.

**FIGURE 6:**
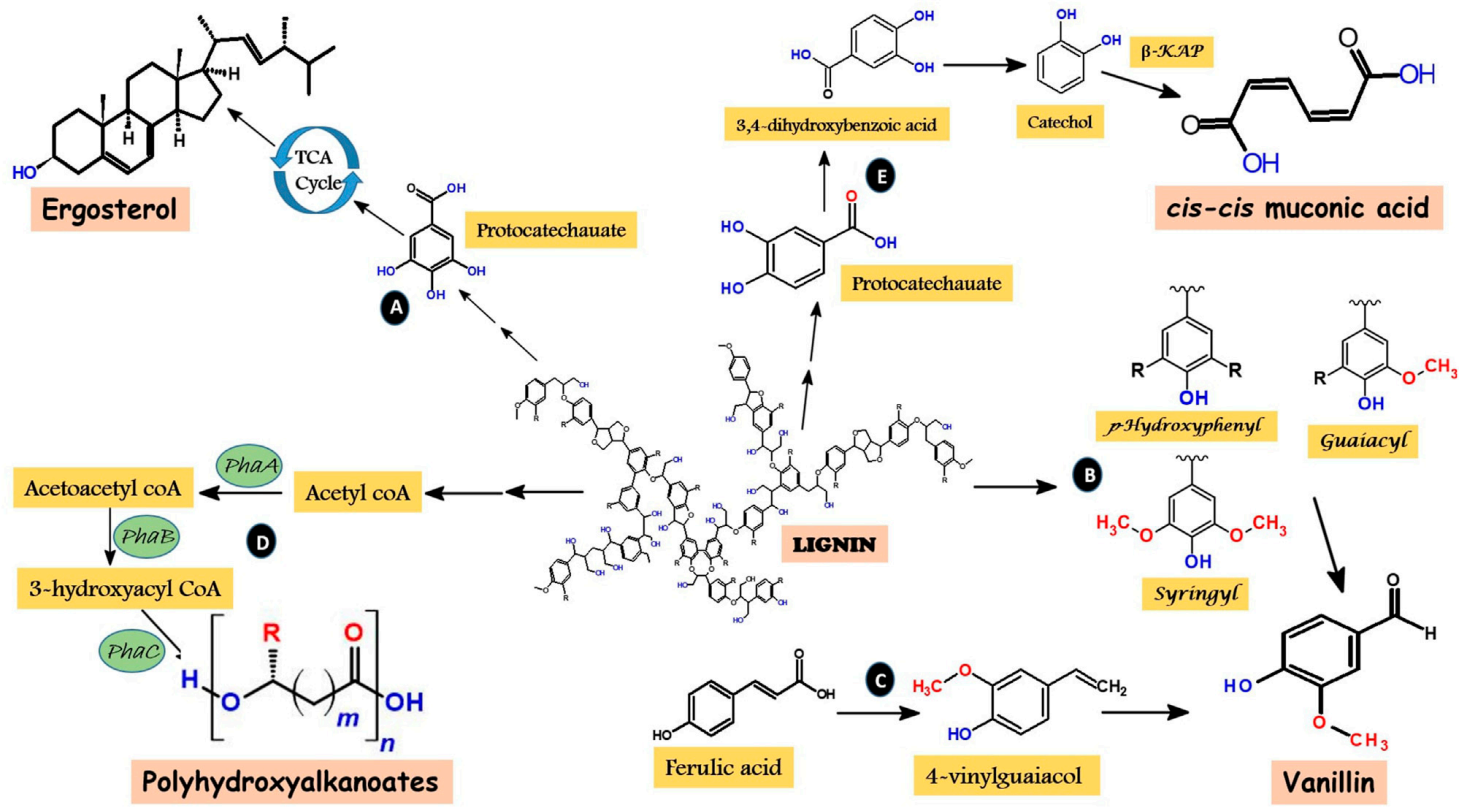
Overview of various pathways involved in selective degradation of lignin into desired valuable products. **(A)** Key route to synthesize microbial lipids, with ergosterol as an example via TCA cycle. **(B)** Vannilin biosynthesis through lignin degradation to monomeric units. **(C)** Vannilin biosynthesis through degradation of ferulic acid. **(D)** Highlights of the multiple pathways in formation of polyhydroxyalkanoates where acetyl CoA acts as the main player. **(E)** Key reactions involved in *cis-cis* muconic acid formation. Where TCA, Tricarboxylic acid; β-KAP, β-ketoadipate pathway.

Endophytes have gained essence on account of their fabulous biotechnological relevance. However, their role as primary colonizers on dead plants has attracted attention due to their lignin deconstruction and bioremediation potential. They are one of the potent sustainable delignifiers capable of producing numerous essential products from the renewable underutilized resource—lignin (Figures 5, 6A–E). Exploration of various endophytic microbes for this potential will not only provide an additional source for the conversion of lignin into such valuable products, but may also unveil novel pathways of conversion with more feasibility in their industrial applications. The most valuable outcomes of delignification are briefly highlighted as follows:

### Microbial Lipids (Single Cell Oils)

Several oleaginous microorganisms are capable of producing lipids or microbial oils exhibiting applications in diverse fields including biodiesel production. Biodiesel is a non-toxic, renewable, and biodegradable fuel which comprises of fatty acid methyl esters. Similar to petroleum diesel, it is used to fuel compression-ignition engines. Currently, its industrial production is mainly dependent on the vegetable oils which can put a par on edible oils resulting in price elevation. Therefore, it is imperative to turn towards the alternative sources for biodiesel production. Interestingly, microbial lipids have attracted a lot of attention in the recent years as an efficient feedstock for biodiesel production. However, their production faces a number of challenges, such as, high costs for lipid extraction, cultivation of microbes, and continuous flux of carbon source (Santek et al., 2018). Intriguingly, utilizing lignocellulosic biomass as a renewable carbon source has effectively reduced the production cost of microbial lipids. Numerous bacteria and fungi are known to produce microbial lipids from lignin. For instance, oleaginous *Rhodococcus opacus* DSM 1069 efficiently used the Kraft lignin to accumulate lipids, mainly palmitic (46.9%) and stearic (42.7%) acids, with the maximum yield of 0.067 mg ml^−1^ after incubation of 36 h (Wei et al., 2015).

Endophytes have great potential to be utilized for the sustainable production of microbial lipids from ligocellulosic biomass. Several studies have unravelled the oleaginous endophytes from diverse plant sources (Zhang et al., 2014; Wani et al., 2017; Das et al., 2022). Both endophytic bacteria and fungi are reported for their oil producing ability (Table 4). *Mucor circinelloides* Q531 isolated from mulberry stem and leaves outstandingly converted mulberry branches into lipids (Qiao et al., 2018). The maximum lipid content of 28.8 ± 2.85% was procured with the yield of 42.43 ± 4.01 mg per gram dry substrate (gds). Further, GC-MS analysis revealed palmitic acid (C16:0, 18.42%), palmitoleic acid (C16:1, 5.56%), stearic acid (C18:0, 5.87%), oleic acid (C18:1, 33.89%), linoleic acid (C18:2, 14.45%) and γ-linolenic acid (C18:3 n6, 22.53%) as the major fermentation products.

**TABLE 4:** Various lipids produced by oleaginous endophytes.

Endophyte	Source	Oils/lipids	References
*Muscodor albus*	*Cinnamomum zeylanicum*	1-butanol, 3-methyl-, acetate	Strobel et al. (2001)
*Microsphaeropsis* *Phomopsis* *Cephalosporium* *Sclerocystis* *Nigrospora*	*Keteleeria evelyniana Mast, Keteleeria davidiana var chienpeii, Cupressus torulosa D.Don, Sabina chinensis* cv. *Kaizuca and Taxus chinensis* var. *mairei, Sabina chinensis, Pinus massoniana*	Oil	Peng and Chen (2007)
*Gliocladiun roseum* NRRL 50072	*Eucryphia cordifolia*	Multiple volatile and non-volatile lipids	Strobel et al. (2008)
*Colletotrichum* sp. and *Alternaria* sp.	*Ocimum sanctum* and *Brassica juncea*	Neutral lipids Phospholipids Glycolipids	Dey et al. (2011)
*Fusarium* sp. ML-GEN.1	*Strobilanthes cusia*	Oleic acid, Palmitic acid Linoleic acid, Unsaturated fatty acids	Xie et al. (2013)
*Bacillus subtilis*	*Juglans regia*	Myristic acid, Palmitic acid Stearic acid, Oleic acid, Linoleic acids	Zhang et al. (2014a)
XF4, XF21, XF36, XF38, XF59, XF72, XF75, XF81, XF89, XF95	*Torreya grandis*	Linoleic acid Oleic acid Sciadonic acid	Yang et al. (2015)
*Bacillus* spp.	*Arachis hypogaea* *Brassica napus* *Brassica nigra* *Helianthus annuus* *Ricinus communis* *Sesamum indicum*	Poly(3-hydroxybutyrate)	Das et al. (2017)
*Ceriporia lacerata* CHZJU	_	Mannosylerythritol lipids (MELs)	Niu et al. (2017)
*Mortierella alpina* CS10E4	*Crocus sativus*	Crocin, picrocrocin and safranal	Wani et al. (2017)
*Fusarium* spp. *Akanthomyces attenuates* *Luteibacter* sp.	*Astrocaryum sciophilum*	2-hydroxy-13-methyltetradecanoic acid, 3-hydroxy-14-methylpentadecanoic acid, β-hydroxypalmitic acid, 3-hydroxy-15-methylhexadecanoic acid, 3-hydroxy-13-methyltetradecanoic acid	Barthélemy et al. (2019)
*Piriformospora indica*	Procured from ICGEB, New Delhi (Mostly endophytic to Barley)	Succinate, oxo-propanoate, l-alanine, glutamate, acetate and 1,2 propanediol, hydroxy butane	Bhatnagar et al. (2019)
*Aspergillus flavus* *Penicillium echinulatum* *Microascus croci* *Penicillium purpurogenum*	*Ascoseira mirabilis* *Adenocystis utricularis* *Desmarestia ancep*s *Phaeurus antarcticus*	Palmitic, linoleic, oleic acid, stearic acid, ergosterol	Teixeira et al. (2019)
*Pseudofusicoccum* sp.	*Annona muricata*	_	Abba and Eze et al. (2020)
*Lasiodiplodia exigua* SPSRJ27 *Phomopsis* sp. SPSRJ28 *Pestalotiopsis microspora* SPSRJL35 *Phomopsis* sp. SPSRJL36	*Jatropha curcas* *Pongamia pinnata* *Sapindus mukorossi* *Mesua ferrea* *Terminalia bellerica* *Cascabela thevetia* *Ricinus communis*	Saturate, aromatic, resin, asphaltene	Paul et al. (2020)
*Bacillus pumilus* AHSD 04	*Arachis hypogaea* L*.*	Poly(3-hydroxybutyrate)	Das et al. (2022)

“_” Not mentioned in literature.

Endophytes are largely cherished for their potential to mimic their host plants in the production of certain essential metabolites, thereby acting as efficient alternatives to conserve these plants in nature. In view of this ability, explorations of oleaginous/biodiesel plants (plants that are traditionally used to produce biodiesel) for the recovery of oleaginous endophytes have gained essence (Strobel et al., 2001; Peng and Chen, 2007; Strobel et al., 2008; Dey et al., 2011). For example, biodiesel plants, such as, *Jatropha curcas*, *Pongamia pinnata*, *Sapindus mukorossi*, *Mesua ferrea*, *Terminalia bellerica*, *Cascabela thevetia*, and *Ricinus communis* proved competent sources for unveiling oleaginous endophytic fungi (Paul et al., 2020). Among the procured oleaginous fungi, the lipid content of *Lasiodiplodia exigua* SPSRJ27, *Phomopsis* sp. SPSRJ28, *Phomopsis* sp. SPSRJL36, and *Pestalotiopsis microspora* SPSRJL35 was 20% more compared to their dry biomass. Similarly, an endophytic bacterium *Bacillus subtilis* HB1310 isolated from thin-skinned walnut effectively used cotton stalk hydrolysate to produce lipids (Zhang et al., 2014). In 48 h of culture time, the lipid content of 39.8% (w/w) with the maximum productivity of 2.3 g/L, and cell dry weight of 5.7 g/L was achieved. Earlier, yeast and microalgae were regarded as the most promising oleaginous agents using glucose or acetic acid as the carbon source (Meng et al., 2009). However, low lipid yields were witnessed with the application of agricultural and industrial wastes as substrate (Santala et al., 2011). In comparison, HB1310, surprisingly, utilized cotton stalk hydrolysate to produce the lipid content comparable to that of yeasts. To have better insights into the lignocellulose conversion efficiency of HB1310, whole genomic information to trace the metabolic pathways has recently been reported (Zhang et al., 2021), which indicated the involvement of Embden–Meyerhof–Parnas, pentose phosphate, and fatty acid synthesis pathways as the major key players in substrate utilization and lipid biosynthesis (Figure 6A). Moreover, higher expression of fatty acid synthesis genes was observed compared to that of other *Bacillus* strains. Tricarboxylic acid (TCA) cycle ruthlessly shared the carbon flux flowing from acetyl CoA node before 48 h and acetic acid fermentation pathway competed after 72 h for the flux distribution of lipid synthesis. Conclusively, endophytes share excellent potential in microbial lipid production from lignocellulosic biomass, and can be used to develop efficient biofuel minifactories. Besides, in view of the abovementioned potential of endophytes, it is pivotal to explore the maximum endophytic microbes for their efficacy in microbial lipid production using lignocelluloses.

### Vanillin (4-Hydroxy-3-Methoxybenzaldehyde)

Vanillin is an outstanding compound which apart from its cosmetic (fragrance) properties act as the precursor for numerous pharmaceutical polymers (Brianna et al., 2016). It can be naturally procured from plants or artificially through chemical synthesis, however, utilizing lignin as the feedstock for vanillin synthesis by microbes confers an eco-friendly, clean and green technology. Vanillin production from delignification has been carried out since 1950 on an industrial scale (Maeda et al., 2018). Initially it was synthesized by aerobic oxidation of sodium lignosulfonate in the presence of alkali NaOH (Forss et al., 1986). However, microbial conversion of lignin to yield vanillin involves a sustainable way to valorize lignin.

Vanillin can either be produced by the lignin depolymerization (Figure 6B), or degradation of ferulic acid. Ferulic acid, a kind of hydroxycinnamic acid is an important organic acid in the cell wall of herbaceous plants, acts as a standard model compound for G-lignin. Few endophytes are known to deconstruct this compound into vanillin. For instance, a bacterium *Enterobacter* sp. Px6-4 colonizing the roots of *Vanilla* plant metabolized ferulic acid into 4-vinylguaiacol and subsequently into vanillin (Li et al., 2008) (Figure 6C). Initially, vanillin was known to be produced from *Vanilla* plants only, however, many endophytic fungi colonizing this plant are known to mimic their host in the production of vanillin. Khoyratty et al. (2022) recently reviewed the vanillin biosynthetic potential of fungal endophytes in *Vanilla* plants to have better understanding of vanillin biosynthesis, bioproduction and biotechnology.

Likewise, *Colletotrichum gloeosporioides* TMTM-13 (an endophytic fungus) isolated from *Ostrya rehderiana* was found with potential to degrade ferulic acid into vanillin, acetovanillone, vanillic acid, and dihydroconiferyl alcohol (Zhang et al., 2019). Similarly, endophyte *Phomopsis liquidambari* used ferulic acid as the sole carbon source and within 48 h, more than 97% of ferulic acid added to mineral salt medium and soil was decomposed (Xie and Dai, 2015). The pathway involved decarboxylation of ferulic acid into 4-vinyl guaiacol followed by oxidation into vanillin and vanillic acid. These compounds upon subsequent demethylation yielded protocatechuic acid which was further degraded via β-ketoadipate pathway. Identification and quantification of metabolites was accomplished adopting GC-MS and HPLC-MS analyses (Xie and Dai, 2015). Interestingly, substrate and product concentrations affected the activities and expression levels of fdcB3 (ferulic acid decarboxylase), lacB3 (laccase), and pcaB3 (protocatechuate 3, 4-dioxygenase), however, transcription of all the three genes was induced in *P. liquidambari*. With the advent of multi-omics technology, more accessibility of metabolic pathways involved in microbial delignification is being achieved (Zhu et al., 2017; Moraes et al., 2018; Zhu et al., 2018).

### Polyhydroxyalakanoates

Polyhydroxyalakanoates (PHA’s) are bioplastics with their promising role in pharmaceutical industry as anticancerous agents, drug carriers, and memory enhancers. Numerous microbes are being reported to produce PHA’s under stressful conditions of nitrogen, potassium, oxygen, phosphorus, and magnesium (Ray and kalia, 2017). Although PHA’s share similar properties with synthetic plastics, their biodegradable, biocompatible, and non-toxic behaviour makes them unique. Diverse microbes are known to produce PHA’s as the degradation product of lignin. For instance, *Pseudomonas* strains have marvellous contributions in lignin valorization via bioconversion of recalcitrant lignin into desirable bioplastics which paves their way for proper place in future biorefinaries. Using lignin as the sole carbon source, *Psuedomonas putida* NX-1 successfully produced PHAs (Xu et al., 2020). Moreover, the physical properties of PHAs liberated from lignin were similar to that synthesized from glucose thereby making lignin-derived aromatics as the efficient alternatives for PHA synthesis. As already mentioned, microbial PHA synthesis is positively correlated to diverse nutrient deficiencies, the biofunneling capacity of *Pseudomonas* strains to yield *mcl*-polyhydroxyalkanoate (*mcl*-PHA) was enhanced after subjected to nitrogen and oxygen limitations (Ramírez-Morales et al., 2021). Recently, conversion of lignin into PHA’s by *Psuedomonas putida* KT2440 was enhanced by the addition of glycerol to the lignin derivatives, such as, vanillin, vanillic acid and benzoic acid (Xu et al., 2021).

In a soil bacterium β-proteobacterium *Pandoraea* sp. ISTKB, lignin degradation and PHA production was witnessed, where Kraft lignin was efficiently degraded into bioplastic PHA’s (Kumar et al., 2018). The genomic and proteomic analysis revealed that various enzymes, such as, laccases, peroxidases, Dyp-type peroxidase, etherases, aldehyde oxidase, dehydrogenases, glycolate oxidase, GMC oxidoreductase, quinone oxidoreductase, dioxygenases, monooxygenases, glutathione-dependent, reductases, and methyltransferases were expressed during the process (Kumar et al., 2018). However, after detailed investigation, three enzyme systems viz., laccases, peroxidases, and Fenton-reaction enzymes came into limelight for their catalytic lignin biodegradation and gene clusters comprising *bktB*, *phaR*, *phaB*, *phaA*, and *phaC* genes to be involved in PHA synthesis (Liu et al., 2019) (Figure 6D).

Many endophytes have also shown their potential for PHA synthesis. Interestingly, the highest yield of PHA’s has been observed using non-conventional carbon sources (lignin-based substances). For instance, an endophytic bacterium *Bacillus cereus* HAL 03 colonizing leaves of Helianthus annuus was found to procude a homopolymer of 3(hydroxybutyric acid)/P(3HB), the most common polyhydroxyalkanoate. Identity of the compound was confirmed by the Fourier-transform infrared and proton nuclear magnetic resonance spectroscopic analysis (Das et al., 2016). Using sucrose (2%) and yeast extract (0.2%) as the carbon sources, P(3HB) production by the bacterial isolate reached 50.46 % and 53.19%, respectively, of its dry cell weight (CDW), while molasses as the carbon source could further scaele-uo the yield upto 54.05% of its CDW. Similarly, another strain *Bacillus cereus* RCL 02 isolated from the leaves of *Ricinus communis* was found to produce poly(3-hydroxybutyrate) [P(3HB)] (Das et al., 2017). Scanning electron microscopy revealed the release of these granules as a function of autolysis by the bacterium. The bacterial isolate produced 68%, 72.2%, and 81% P(3HB) of its CDW when grown in glucose with mineral salts, glucose with yeast extract, and metal stress (1.5 mM manganese), respectively. However, the polyester production was further enhanced to 83.6% CDW after using refined sugarcane molasses as the sole source of carbon. Bacteria, such as, *Azospirillum*, *Burkholderia*, and *Herbaspirillum* isolated as nitrogen fixing endophytes from *Asparagus officinalis* were also found to accumulate PHA’s in their cytoplasm (Altamirano et al., 2021).

### Muconic Acid (C_6_H_6_O_4_)

It is a diacrboxylic acid with three stereoisomeric forms—cis-cis muconic acid, trans-trans muconic acid and cis-trans muconic acid which differ in spatial arrangement of atoms/functional groups around the double bonds. *cis-cis* muconic acid is mainly produced by microbial degradation of aromatics and find its applications in making polyurethane, polyethylene terephthalate (PET), and nylon (Curran et al., 2013; Wang et al., 2020). Several microorganisms are reported to produce cis-cis muconic acid as an intermediate in lignin biodegradation (Wang et al., 2020). However, recently, metabolically engineered microbes for the selective conversion of lignin into cis-cis muconic acid has gained essence (Pyne et al., 2018; Choi et al., 2020; Aravind et al., 2021; Wirth and Nikel, 2021). Most of the microbes convert lignin into *cis-cis* muconic acid by using glucose as the additional growth substrate (Becker et al., 2018). However, engineered *Pseudomonas putida* KT2440 strain converted vanillic acid (guaiacol-based lignin model) and 4-hydroxybenzoic acid (p-hydroxyphenyl-based lignin model) into muconic acid (Sonoki et al., 2018). More interestingly, while KT2440 could yield 20% of *cis-cis* muconate, *Sphingobium* sp. SYK-6 yielded 45% after using syringic acid as the lignin-based model compound.

As far as endophytic delignification to yield muconic acid is concerned, very few reports are published so far. For instance, during an investigation, endophytic *Phomopsis liquidambari* was evaluated for its potential to degrade 4-hydroxybenzoic acid (4-HBA) as it comprises one of the phenolic allelochemicals produced after foliage decomposition. Intriguingly, the endophyte used 4-HBA as the sole carbon source thereby led to its degradation into *cis-cis* muconic acid (Chen et al., 2011). Unravelling the metabolic pathways following high performance liquid chromatography–mass spectrometry (HPLC–MS) and gas chromatography–mass spectrometry (GC–MS), 4-HBA first undergo hydroxylation to form 3,4-dihydroxybenzoic acid and then catechol followed by oxidation to *cis-cis* muconic acid in the tricarboxylic acid cycle (TCA) cycle. Upon feeding with ferulic acid as the sole carbon source, *P. liquidambari* produced vanillin and vanillic acid, as already mentioned under “Vanillin” sub-section. However, these intermadiates undergo demethylation yielded protocatechuic acid which was further degraded via β-ketoadipate pathway to form β-carboxy-cis, cis muconic acid (Xie and Dai, 2015) (Figure 6E). Research is essentially lagging regarding the application of endophytes in lignin depolymerization to yield such dicarboxylic acids. Therefore, various intermediates produced after lignin depolymerization/pre-treatment can be funnelled to produce muconic acid.

### Optimization in Endophytic Delignification

#### Hybrid Technology

Since biological lignin depolymerization is very slow, hybrid technology is being introduced which functions by combining chemical and biological depolymerization. For example, hybrid biochemical routes were applied for the formation of desired products where lignin was first chemically depolymerized into vanillin and syringate as chief products. With the application of engineered *Escherichia coli* strains, these products were subsequently bio-converted into *cis*, *cis*-muconic acid (ccMA) and pyrogallol (Wu et al., 2017).

Chemically depolymerized lignin biopolymer yields heterogenous compounds which are further converted into common products by biological funnelling (Linger et al., 2014). Considering the production of desired products from lignin degradation, integrated biocatalysis is being employed. For instance, *Rhodococcus opacus* PD630, a strain capable of utilizing multiple lignin-derived aromatic compounds for its growth (Xie et al., 2019), was rationally designed to produce single aromatic compound, gallate (3,4,5-trihydroxybenzoic acid) (Cai et al., 2021). Gallate is widely used in food, cosmetic, and pharmaceutical industries (Badhani et al., 2015; Fernandes and Salgado, 2016). Thus, target product (gallate) was produced by coupling three main reactions—aryl side-chain oxidation, hydroxylation, and O-demethylation in *R. opacus* PD630 by enhancing endogenous pathways and establishment of exogenous biocatalytic systems. Essentially, alkaline-pretreated lignin and base-depolymerized ammonia fiber explosion (AFEX) lignin constituents were more efficiently converted into gallate in single reactor in comparison to the traditional multi-reactor based technology.

Recently, an efficient strategy was unveiled employing a novel laccase producing endophytic fungus *C. globosum* (colonizing flower of *H. manihot* L.) in combination with ultrasonic waves to improve lignin degradation in larch sawdust (Dou et al., 2022). Compared to individual estimates, rate of delignification enhanced upto 34.29% which corresponds to 1.5-fold and 2-fold elevation by fungus and ultrasound, respectively. Intriguingly, out of three monomeric lignin units, p-Hydroxyphenyl and Guaiacyl increased from 0.46 to 0.70 mg/g while significant increase was not witnessed in Syringyl propane, indicating the selective nature of endophytic laccase.

#### Microbial Consortia

To overcome the limitations with single microbial strains and other shortcomings of traditional delignification, a group of microbes is used which act synergistically to degrade lignin biopolymer. In a comparative analysis, pre-treatment of tree trimmings with fungal monocultures and microbial consortia was evaluated for the selective degradation of lignin. Interestingly, microbial consortia DM-1 decomposed 14.0% of lignin with no significant cellulose degradation for initial 20 days (Lin et al., 2020). Besides, the SSF (simultaneous saccharification and fermentation) method revealed 20% increment in ethanol production with DM-1 treated microbial consortia as compared to control (Kanagasabai et al., 2019). Similarly, pre-treatment by a novel microbial consortium was investigated for its influence on methane production (Raut et al., 2021). In this study, microaerobic barley straw-adapted microbial (BSAM) consortium was used for the pretreatment of Barley straw and co-substrate hay followed by anaerobic digestion. Surprisingly, BSAM pre-treatment yielded methane content of 58% (v/v) in total biogas produced in comparison to 10% (v/v) of control.

Compared to single strain-based techniques, microbial consortia shows significantly higher expressions of ligninases thereby elevates the yield of value-added products. For instance, mixed bacterial culture was found to be more efficient in converting Kraft lignin into vanillin (Baghel and Anandkumar, 2019). Similarly, laccase (Lac) and Manganese peroxidase (MnP) activity of *Lenzites betulina* and *Trametes versicolor* was enhanced by 40% after taking a consortium as compared to monocultures under the same culture conditions (Cui et al., 2021). In another extensive study, out of several screened consortia from wooden antiques for lignocellulose deconstruction, four different consortia (J-1, J-6, J-8 and J-15) exhibited degradation ability of lignin. With an initial lignin concentration of 0.5 g/L at pH 4 and rotation speed of 200 rpm, the catalytic efficiency of J-6 reached 54% after 48 h (Zhang et al., 2021b). The main fungal components of consortium J-6 comprised of Saccharomycetales (98.92%), followed by Ascomycota (98.92%) while bacterial components of Shinella sp. (47.38%), *Cupriavidus* sp. (29.84%), and *Bosea* sp. (7.96%). Saccharomycetes might possessed high adaptability to the system with potential to enhance enzymatic activities while abundant bacteria accelerated the depolymerisation. Thus, it is imperative to unveil the fungal and bacterial strains with effective lignin-utilization capacity. In this context, few researchers used natural selection over time to screen bacterial strains capable of using lignin as the sole source of carbon (Mendes et al., 2021).

Interestingly, in a study by Patil et al. (2016), instead of utilizing consortia of isolated microbes, an attempt was made to couple the efficacy of plant organs with their isolated endophytic microbes. For example, 20 ppm of Navy Blue HE2R (NB-HE2R) was decolourized by *in vitro* grown adventitious roots (AR) of *Ipomoea hederifolia* and its endophytic fungus (EF) *Cladosporium cladosporioides*, respectively, within 96 h. However, AR-EF consortium decolourized the same concentration of HE2R with the efficiency of 97% within 39 h only. Enthrallingly, efficacy elevation was correlated with the induction of laccase, lignin peroxidase, and tyrosinase in AR while laccase, riboflavin reductase, and tyrosinase in EF.

Apart from augmentation in ligninase producing potential and simultaneous synthesis of multiple ligninases, microbial consortia comparatively exhibited more adaptability to varied environmental conditions. A microbial consortium obtained from anaerobic digested sludge showed higher lignocellulose degradation ability of corn stalk under thermophilic conditions as compared to that in mesophilic conditions (Lu et al., 2019). Metagenomics and transcriptomics-based approaches have also gained essence in screening efficient microbial consortia, some of which showed selectively higher degradation rates for lignin (Jiménez et al., 2016; Diaz-Garcia et al., 2020). In an exciting analysis, laccase encoding gene, *lacz1* was screened from macrotranscriptome of a microbial consortium WSC-6 found to degrade lignocellulose (Zhang et al., 2021c). Compared to initial degradation, analysis of *lacz1* via reverse transcription-quantitative PCR (RT-qPCR) revealed enhancement in expression upto 30.63 times in consortium WSC-6 Moreover, this bacterial laccase could withstand high temperature conditions. Therefore, laccase activity of a microbial consortium increases potentially with instances of retrieving stable enzymes.

#### Irradiation Methodology

Previously, laccase and dye decolourizing activity of microbes (endophytes) was enhanced by the supplementary mediators, as already mentioned. Recently, electron beam radiations are being used to increase this ability without the addition of mediators (Navada et al., 2018). For instance, laccase enzyme from an endophytic fungus *Phomopsis* sp. biodegraded and detoxified the recalcitrant dye anthraquinone and Remazol Brilliant Blue R (RBBR) within 5 min. Laccase production was enhanced to 1.6-fold upon irradiating the fungus with electron beam which was found to be less in unirradiated fungus (Navada et al., 2018). During this investigation, an interesting observation was the elevated melanin (dark-brown pigment) production in the *Phomopsis* culture. Melanin is mainly produced by dark-septate fungi which protects these microbes from radiations (Grishkan, 2011). Many studies have revealed the correlation and dependency of laccase genes with melanin biosynthesis (Castro-Sowinski et al., 2002; Lu et al., 2017; Oh et al., 2021). Therefore, radiations trigger the genes encoding melanin which ultimately leads to increased expression of the laccase producing genes (Navada et al., 2018). Furthermore, the laccase produced by irradiated fungus exhibited extreme tolerance to heavy metals, such as, Ca^2+^, Cu^2+^, Cr^2+^, and Zn^2+^ up to 10 mM concentration. The degradation products of chemical depolymerisation of lignin are often toxic. However, the dye products degraded by *Phomopsis* sp. were non-toxic to plants and micro-organisms. LC-MS data analysis indicates degradation of dye into non-toxic molecules by laccase (0.2 kGy) following deamination, hydroxylation, oxidation, and ring cleavage. Similarly, degradation of aniline blue (triphenylmethane dye) was enhanced by 2-fold when the laccase producing endophytic fungus *Phomopsis* sp. was irradiated by gamma rays without mediators (Navada and Kulal, 2020). Furthermore, the degradation products were also non-toxic.

One of the interesting facts is that endophytic microbes are the symptomless colonizers of the plants, till the time any kind of stress prevails (Tanaka et al., 2006; Bamisile et al., 2020). Upon experiencing variable stressors (nutritional, temperature, salinity, etc.), few of them, especially fungi switch their lifestyle from mutualism/commensalism/latent pathogenism to saprophytism or pathogenism (and degrade lignocellulosic biomass) (Deshmukh et al., 2006; Kogel et al., 2006). This stress-switching ability can be used to induce/and enhance the delignifying ability of microbes which must be accompanied with the activation of genes coding for ligninolytic enzymes. Since irradiation is a kind of stressor, it is probable that enhanced delignifying potential of abovementioned endophytes pertains to their stress response-strategy. Therefore, variable stressors can be employed to elevate the lignin deconstruction ability of endophytes.

#### Genetic Engineering/Recombinant Ligninases

Ligninolytic enzyme production in microbes is widely being enhanced by manipulating their genomes. For instance, wild type *Boletus versicolor* (Bv IBL-04) produced laccase with activity of 118.89 ± 11.32 U/ml. A mutant (Bv EB-75″) was synthesized via random chemical mutagenesis with the laccase production of 403.34 ± 13.79 U/ml (Khalid et al., 2020). Apart from using the whole microbes, ligninase producing genes are cloned in suitable vectors and transformed into new hosts for rapid multiplication and transcriptomic analysis (Preethi et al., 2020). Else, certain metabolic pathways of the ligninolytic microbes are being engineered to enhance their degradation ability at variable conditions. For instance, *Bacillus stratosphericus* BCMC2 was isolated from the hindgut of a fungus feeding termite *Macrotermes barneyi* (Xiao et al., 2021). Its laccase gene (BaCotA) was cloned and overexpressed in *E. coli*. Recombinant BaCotA exhibited enhanced lignolytic activity and was thermotolerant at 70°C & pH 5.0 with specific activity. Furthermore, it exhibited tolerance to alkali and organic solvents. Therefore, apart from elevating the efficacy of ligninases with enhanced production of valuable products, recombinant technology improves the specificity and tolerance of enzymes to diverse factors.

Sometimes, coupling of exogenous systems to the existing endogenous pathways also work. For instance, selective synthesis of gallate was accomplished from lignin components by the introduction of an engineered biocatalyst (Cai et al., 2021). Here, aryl side-chain reaction, hydroxylation, and O-demethylation was coupled to enhance the yield of gallate. Initially, sensibly designed hydrolysate system was launched into the gallate biodegradation pathway for the efficient conversion of protocatechuate and upper pathway intermediates into gallate. Secondly, native O-demethylation was harnessed to convert multiple lignin-derived methoxy aromatics into gallate. Lastly, to broaden the substrate spectrum, aryl side-chain reaction was introduced. In this way, rationally designed metabolic engineering is pivotal to improve the lignin valorization ability of microbes into desirable products. Details regarding the importance of engineered microbes in bioconversion of lignocellulose into valuable products has recently been reviewed (Bugg et al., 2021).

### Limitations and Challenges of Endophytes in Lignin Valorization

Endophytes hold a prodigious position pertaining to their applications in diverse fields, viz., agriculture, bioremediation, biotransformation, and industry thereby showcasing their potential in fulfilling various demands in the international market to boost the global economy. Despite offering safe and sustainable alternatives to harmful chemicals, research on endophytic delignification is essentially meagre (Adeleke and Babalola, 2022). This is because endophytes are the cryptic creatures of nature that reside silently within the plant tissues. To check their ligninase ability, endophytic isolation from such microhabitats is imperative which adds on a laborious step towards lignin valorization.

Based on the reports mentioned earlier, endophytes are the treasure hunt for ligninases. However, as mentioned earlier, one of the main concerns involves the low enzyme production by endophytic microbes which remains the major challenge for their industrial applications. Apart from secreting lesser amount of enzymes, certain endophytes are extremely slow growing which further delays the enzyme secretion. Therefore, it is pivotal to boost their enzyme secretion employing modern techniques, like, cell factory engineering and CRISPR (clustered regularly interspaced short palindromic repeats)-Cas (CRISPR associated protein) technology (Raghav et al., 2022). Neverthless, such modifications may hike the overall cost for synthesizing endophytic enzymes. Due to overwhelming incredibility and demands of enzymes, the global market value of enzymes is expected to hike at a compound annual growth rate of 6.1% until 2027, which was USD 5.6 billion in 2019 (Fungal Enzymes Market Report, 2021). Hence, for the maximum utilization of endophytic enzymes and their contribution in enhancing the global economy, the bottlenecks regarding the endophytic growth, purity, maintenance/sub-culturing, and enzyme secretion should be addressed (Tiwari and Bae, 2022). Research is required to explore the ways which could overcome these hurdles without enhancing the production costs.

Besides, endophytes require more energy to break the bonds in recalcitrant lignin polymer owing to the high bond dissociation enthalpies in its functional groups. Therefore, one of the main barriers in the development of endophytic biorefinaries lies in the pretreatment technology. So far, it has been challenging to develop an efficient and cost-effective pretreatment technology for lignocellulose fractionation (Zhao et al., 2022). Another problem lies in the condensation/repolymerization of lignin which requires the application of chemicals, like, aldehydes to uncondense the biopolymer (Amiri et al., 2019). However, to attain the principle of green chemistry, there should be certain green alternatives to these chemicals.

Endophytic microbes exhibit complex relationship with their host plants based on multivariate cross-talks (Mattoo and Nonzom, 2021). Accordingly, they may change their behaviour, growth, and activity. Geographical, physiological, and seasonal variations may also contribute to this change (Fang et al., 2019). Also, after growing in artificial medium for multiple generations, endophytes mainly undergo attenuation which either reduce or completely stop the synthesis of secondary metabolites (Jamwal and Gandhi, 2019). Attenuation of function may similarly work for enzyme production in the artificial medium where endophytes may lose their ability to produce enzymes outside the host plant after a few generations. More so, epigenetic modulators or other inhibitors may work antagonistic to this attenuation. Therefore, it is pivotal for scientists to understand this dynamism and provide host-like conditions in order to trigger the ligninolytic genes, reverse the attenuation, thereby harness the maximum potential of endophytes. Specifically, detailed molecular mechanism of endophytic ligninase producing pathways must be unveiled (Bhadra et al., 2022). Although biological funnelling provides an outstanding natural machinery for lignin bioconversion, complete mechanism of the possible routes involved needs to be unravelled for the effective synthesis of value-added products from lignin. In general, to harness the endophytic bioresouce efficiently, more research is required to overcome the limitations and meet the challenges which currently pose hinderence in the way of its large scale utility.

## Conclusion and Future Perspectives

Lignin valorization is facing multifaceted challenges owing to its structural complexity and heterogeneity which hinders the production of desirable and specific outcome. Thermochemical processes may have certain advantages, like, higher rate of reaction and lesser time requisition, yet high energy requirements and usage of excessive chemicals downgrade the environmental quality. Focussing upon the SDG’s, abundant aromatic resource—lignin needs to be valorized into biofuels and chemicals employing eco-friendly techniques. Using biological agents in valorizing lignin can overcome maximum drawbacks of the thermochemical techniques. However, we are lagging to unravel the diverse pathways that microbes use for lignin deconstruction. For efficient valorization, microbes from various sources are required to be investigated for their delignification efficacy. Endophytes decipher a novel and promising source for lignin valorization on account of their marvellous ability of degrading lignin into valuable compounds. One of the special characteristic features of endophytes being their mimicking ability of essential host metabolites. Since vegetables/plants are exploited as the biggest sources for biofuel production, microbes endophytic to these plants mimic their hosts in the lipid production. Switching to the endophytic lipid production has lessen our dependency on these biodiesel plants, thereby helping in their conservation. As endophytes are least explored for their lignin degrading efficiency, there is much to know about their conversion abilities. It is known that these microbes are more vigorous than white rot fungi in degrading lignin (Fillat et al., 2017). Therefore, various pathways and the mechanisms involved behind this potency need to be unveiled. Also, it is probable that ligninases from endophytes colonizing extremophilic plants may possess extremophilic activities which will make them competent for usage at industrial scale. However, studies are completely lacking in this regard.

Endophytes switch their lifestyle under stressful conditions to saprophytic or pathogenic extremes, this is an indication that endophytes can release ligninases under stressed environments, and artificial stressful conditions provided may enhance their potential to produce ligninases. More so, considering their marvellous delignification potential and underexplored nature, endophytes can be investigated for novel catabolic pathways which could pave way for the new routes to overcome the hurdles in lignin utilization. One of the major issues with the enzymatic hydrolysis of lignocellulose includes non-productive adsorption of enzyme onto lignin caused by the hydrogen bonding, hydrophobic, and electrostatic interactions (Yarbrough et al., 2015). The steric hindrance caused by the non-productive binding of cellulase onto the lignin blocks the effective hydrolysis. This problem can be solved by pre-treating the lignocellulose for the separation of lignin from the other components. It will not only increase the accessibility of surface area of the substrate to cellulase, but also transform the lignin for effective enzymatic hydrolysis. Pre-treatment with thermochemical methods followed by enzymatic depolymerization (hybrid technique) is being employed to overcome this drawback alongwith increasing the rate of reaction. However, hybrid technology will again compromise with the environmental quality. Hence, in-depth investigations are required to unravel eco-friendly optimization techniques to fasten the reaction rate. Genetic engineering and endophytic consortia may prove better optimizers in enhancing lignin depolymerization in an eco-friendly manner. Maximum research is required to unveil the genes involved in endophytic lifestyle switching from mutualism/commensalism/latency to saprophytism, and lignin degradation. Divulging into complete mechanism behind the process and manipulating these genes through recombinant technology can open new vistas of research in improving lignin valorization.
